# Supplementary figures and images for: Probabilistic model based on circular statistics for quantifying coverage depth dynamics originating from DNA replication
Source: PeerJ. 2020 Mar 27;8:e8722. doi: 10.7717/peerj.8722 (PMC7104724; doi:10.7717/peerj.8722)

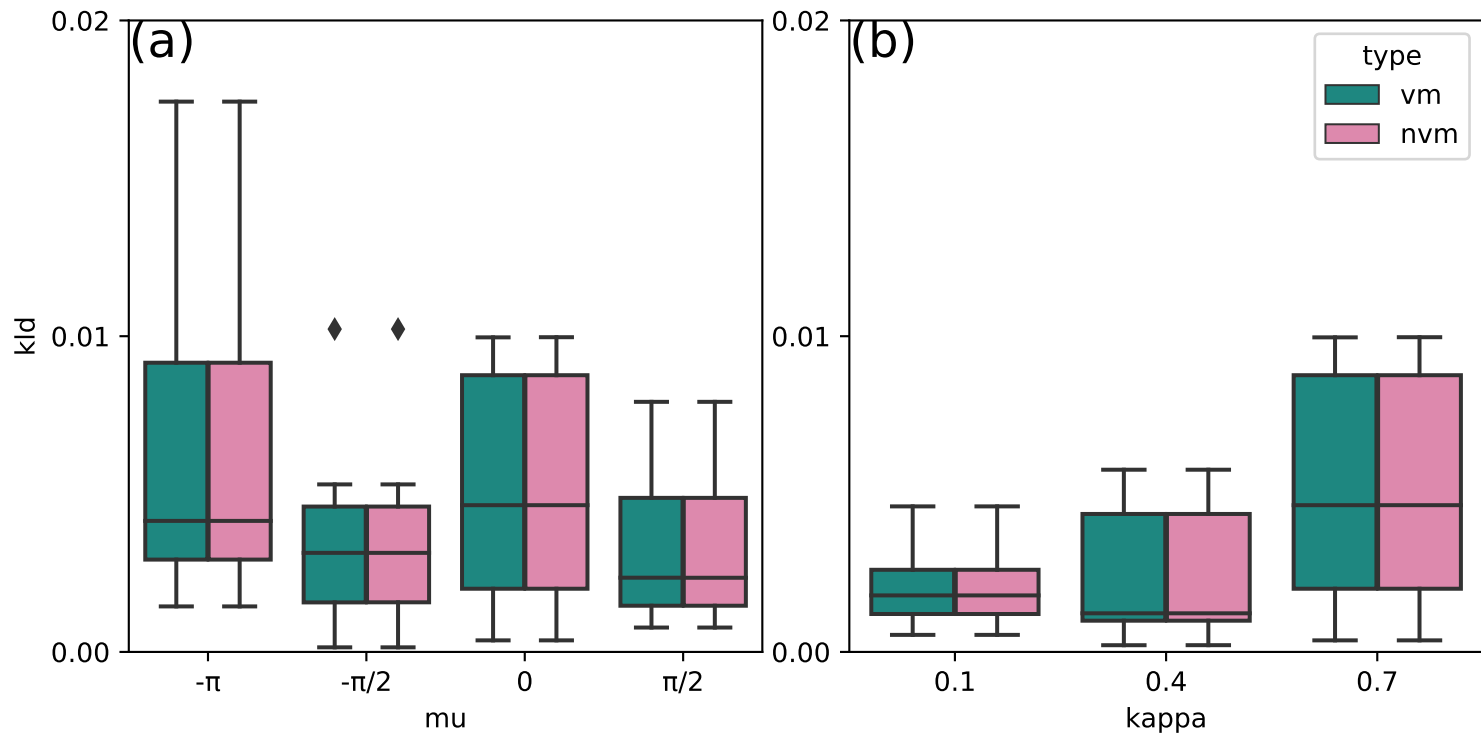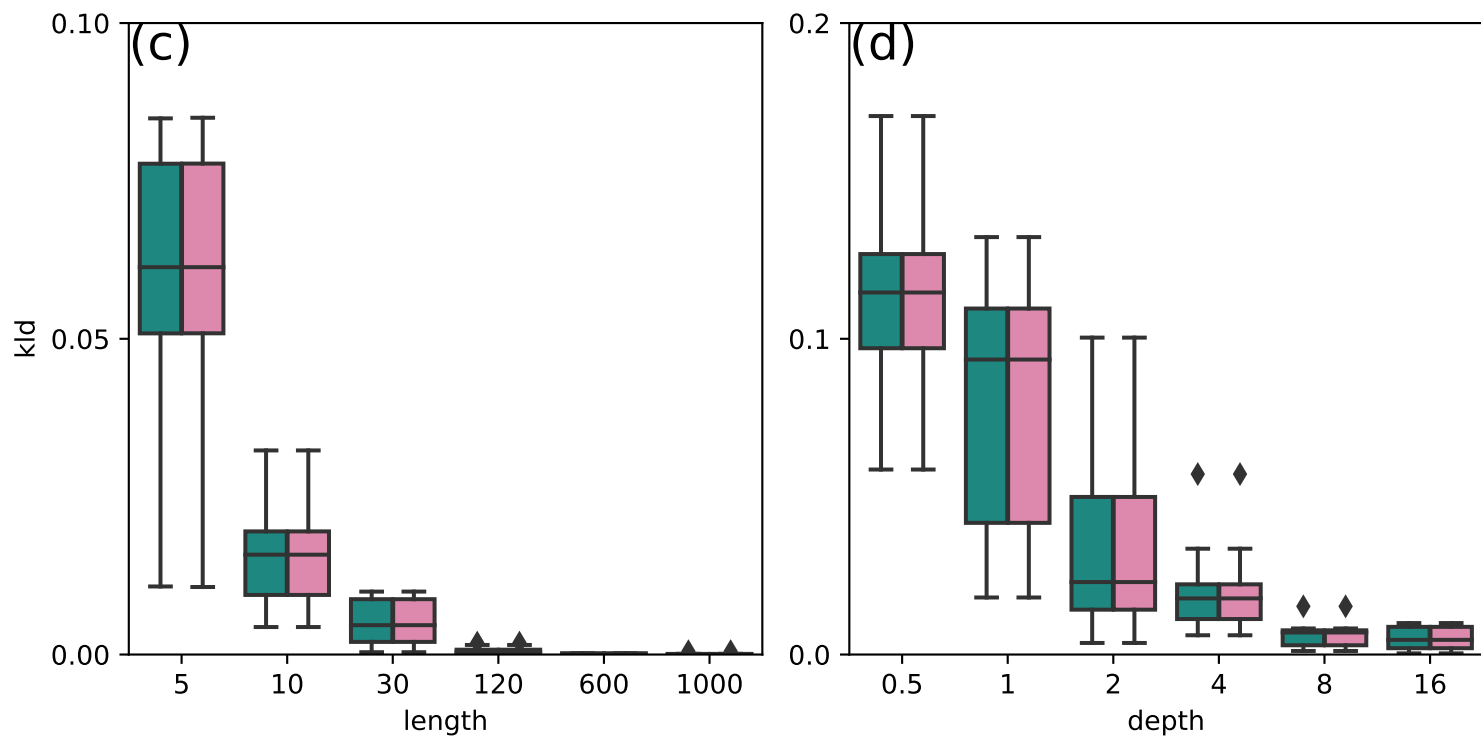

Supplement: Figure S1 — We fitted the unnormalized and normalized von Mises models to a simulation dataset to evaluate the error with respect to the true value. The vertical axis represents the Kullback-Leibler divergence with respect to the true distribution (kld), and horizontal axes indicate the parameters. The green bars indicate the unnormalized von Mises distribution-based model, and the pink bars represent the normalized von Mises distribution-based model when the (A) location parameter was changed, (B) the concentration parameter was changed, (C) the discrete length was changed, and (D) and average coverage depth were changed. [file peerj-08-8722-s001.pdf]

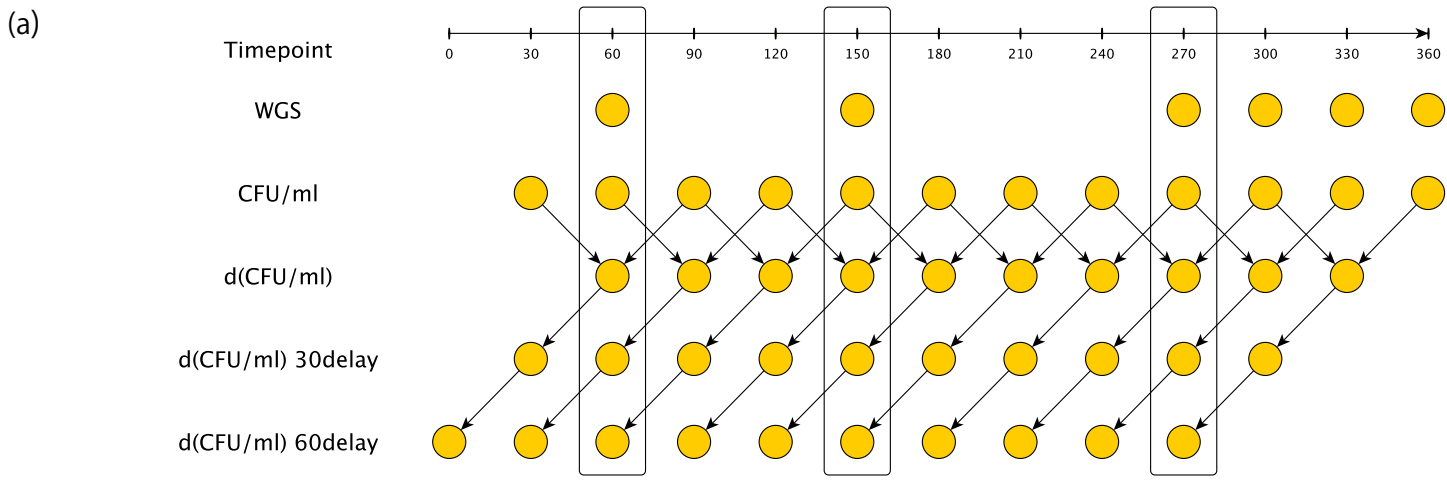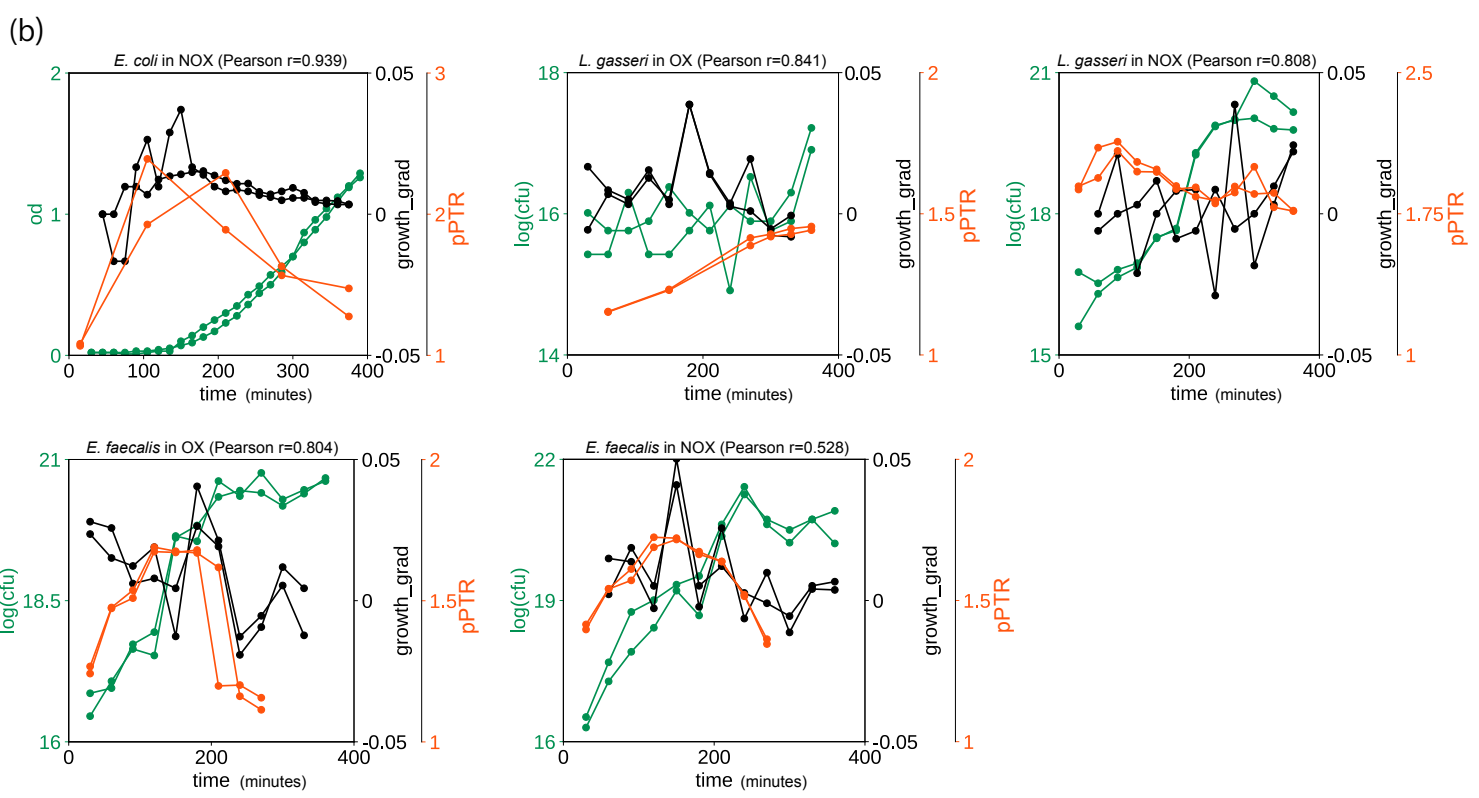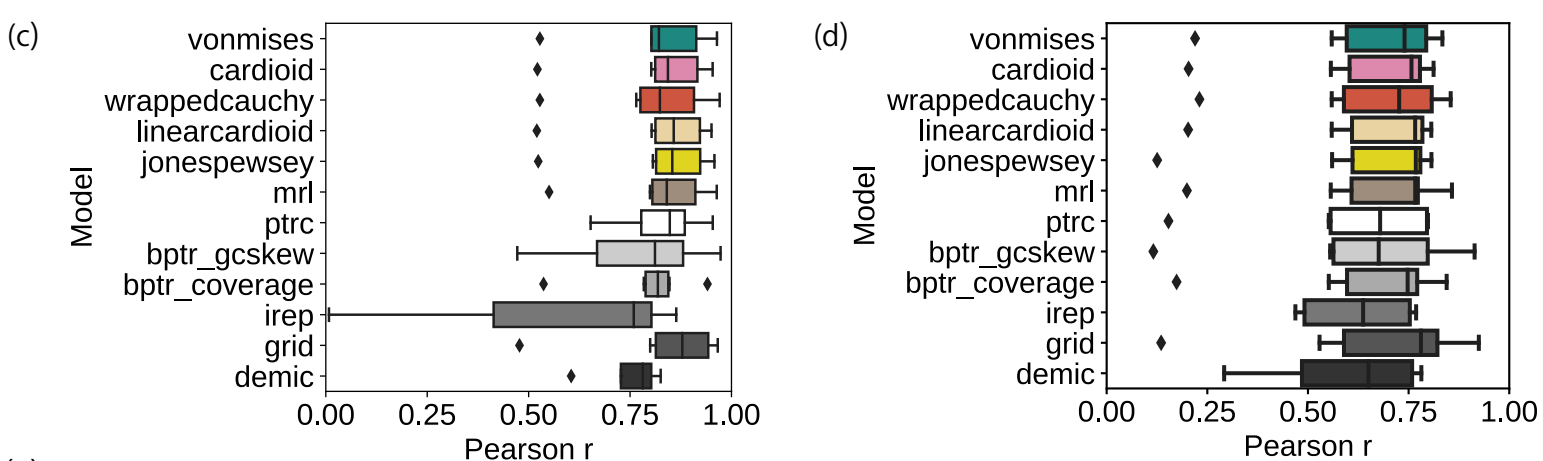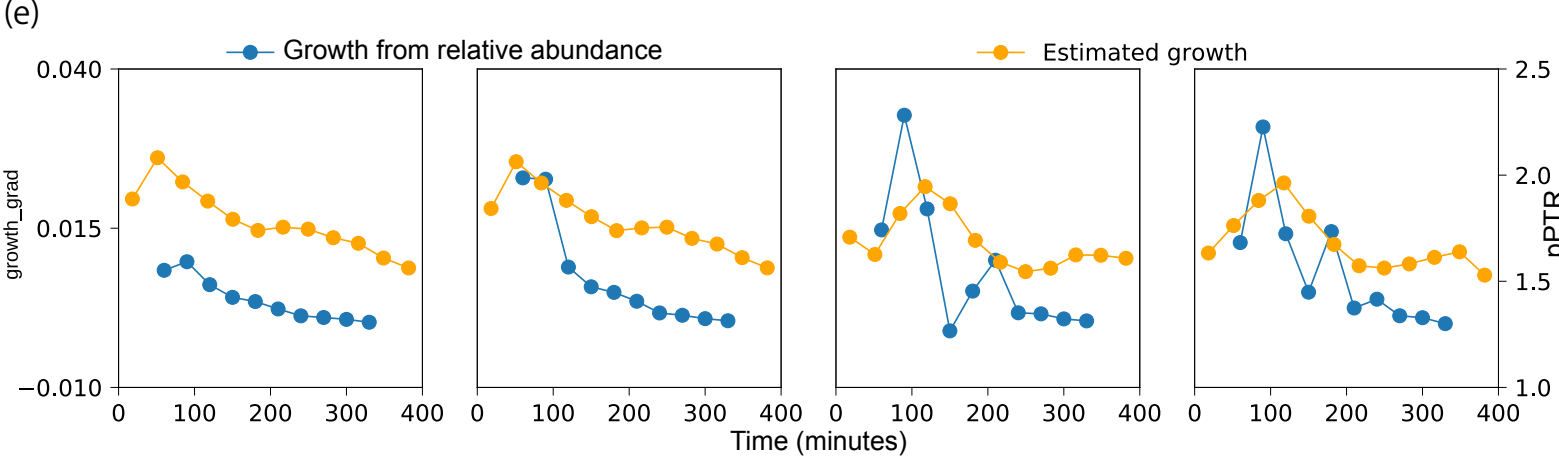

Supplement: Figure S2 — We evaluated the accuracy of the method using experimental growth rates. (A) Method of computing the correlation between the growth estimates from the WGS and the growth rates experimentally obtained from CFU/ml in the L. gasseri OX culture. Considering the delay of cell division due to DNA replication, the growth estimates were shifted before computing the correlation coefficients. The yellow circles represent the existence of data, and the arrows represent the inheritance of values. (B) Growth dynamics according to the experimental growth rates and estimates from the von Mises distribution model. The parameter of the median filter was 100 bp in both cases. The Pearson correlation was calculated separately with shifts for the L. gasseri OX culture (60 min) and NOX culture (120 min). (C) Comparison of the accuracy of this method with those of previous methods. The evaluation was conducted using the experimental growth rate computed according to Korem et al. and (D) using a differential approach to investigate the short-time dynamics. (E) Evaluation of the correlation using a mixed community dataset containing seven species. A high correlation is observable for L. gasseri (r = 0.76 ± 0.04) over four biological replicates. Each panel exhibits a trend in the growth estimate and experimental growth rate in the single replicate from A to D. [file peerj-08-8722-s002.pdf]

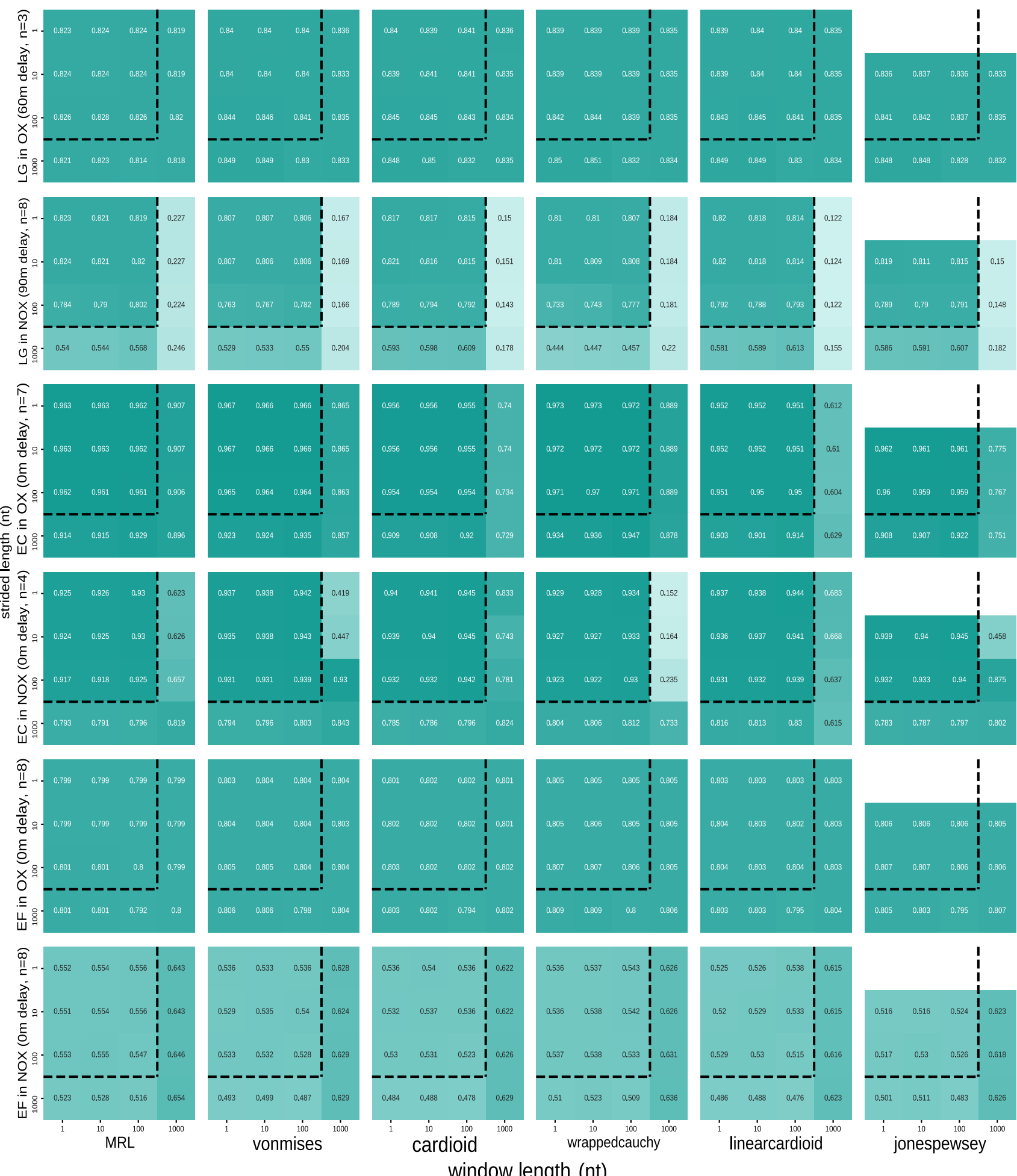

Supplement: Figure S3 — We calibrated the window and stride lengths in the median filter to evaluate their effects on the PTR estimation. The Pearson correlation coefficients between the estimated PTRs and experimental growth rate were obtained using different median filter parameters. We compared the PTRs using MAP estimation. Except for the E. faecalis dataset, a high correlation with the experimental growth rate (≤100 nt for both parameters) was maintained. The same files were used to compute the PTR for the same median filter parameter set in multiple models. The sample size n represents the time point used to compute the correlation coefficient. [file peerj-08-8722-s003.pdf]

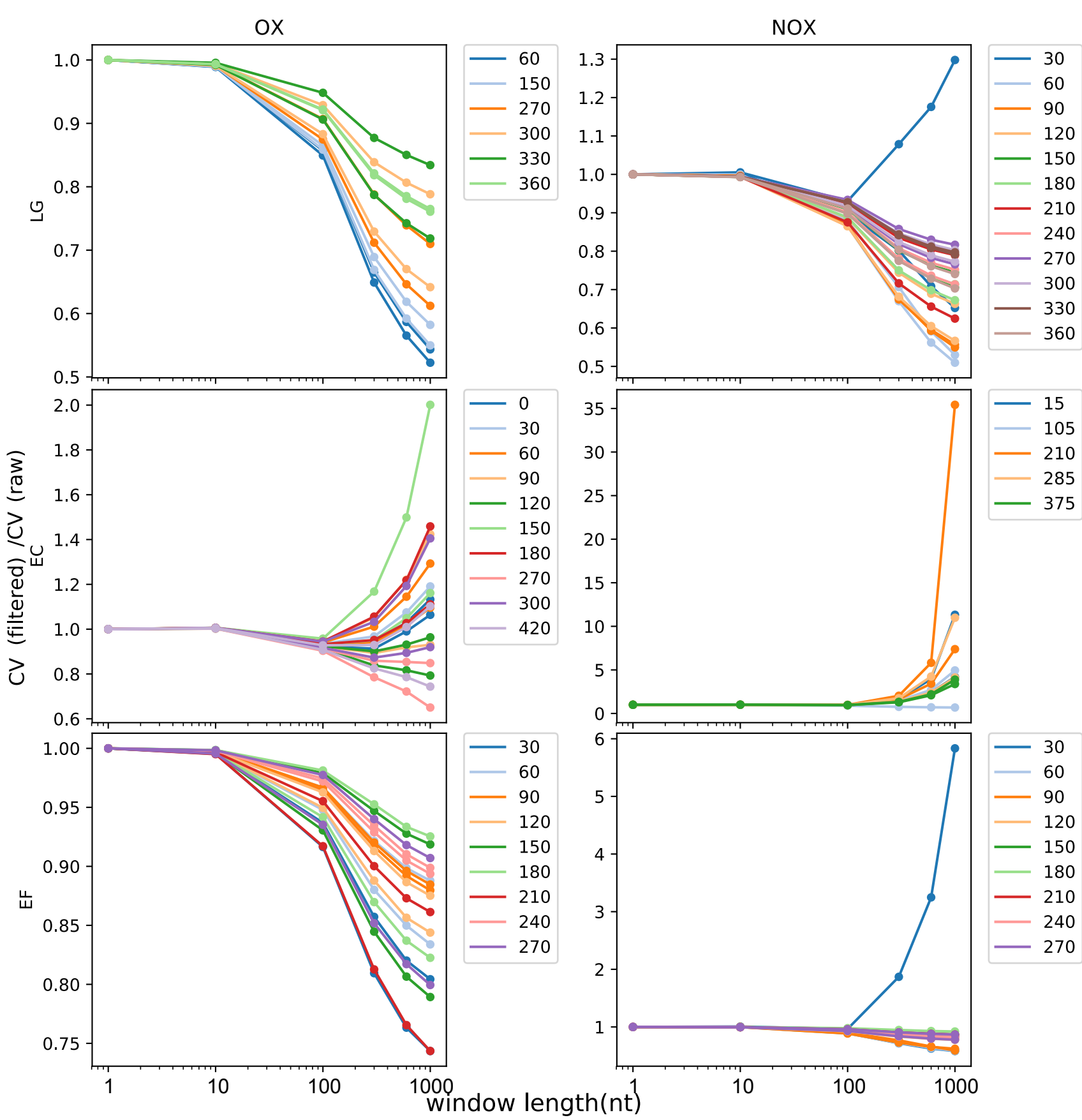

Supplement: Figure S4 — Effect of changing the window length in the median filter on the coverage depth. The y-axis represents the ratio of the coefficient of variance after the application of a median filter prior to filtering. [file peerj-08-8722-s004.pdf]

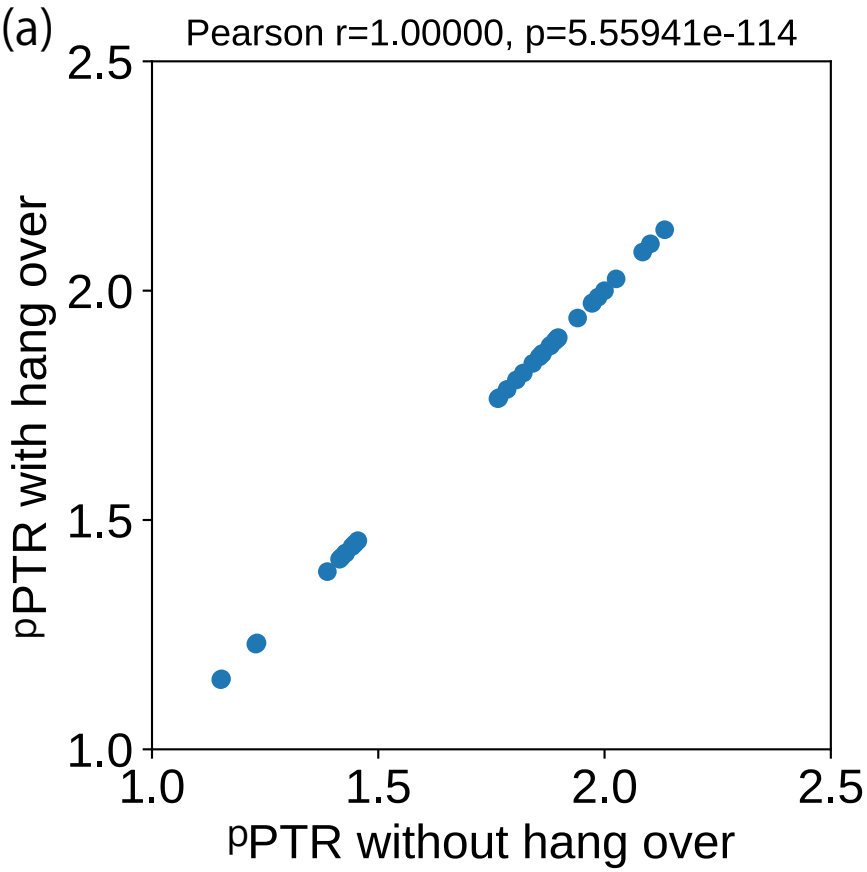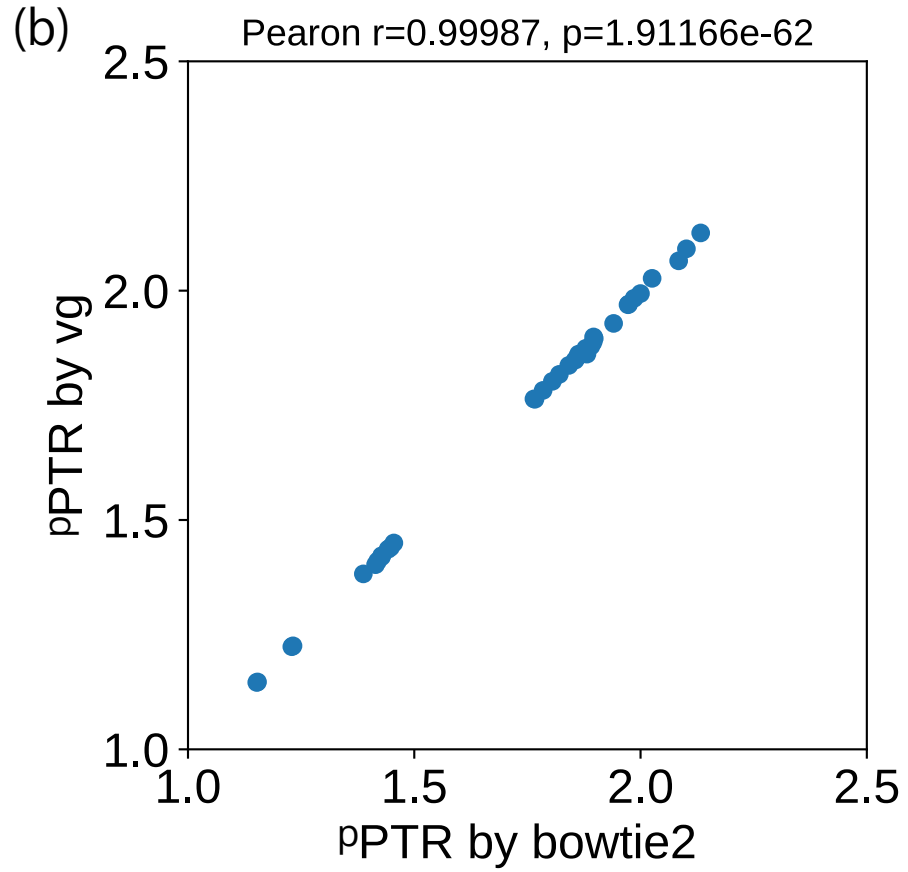

Supplement: Figure S5 — To evaluate the effect of the coverage decreasing at the edge of a sequence, we compared (A) the PTR from the raw sequence with that of the hang-over reference sequence and (B) the PTR obtained using Bowtie2 with that of vg. Both comparative evaluations were performed using a von Mises distribution-based model and the L. gasseri WGSs from Korem et al. [file peerj-08-8722-s005.pdf]

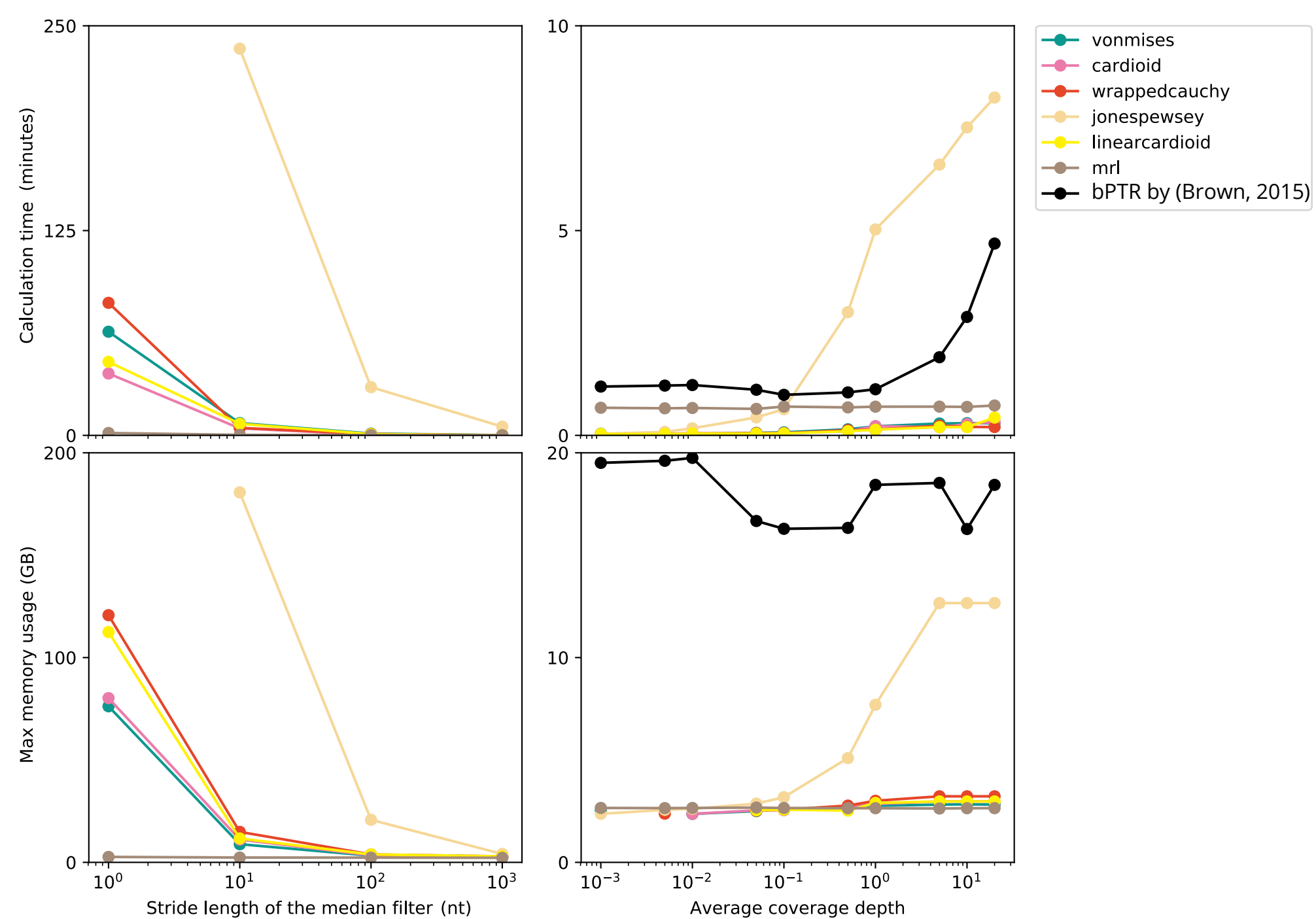

Supplement: Figure S6 — The figures on the left represent the required CPU time and maximum memory usage when the stride length of the median filter was varied, and the window size was fixed to 100 nt. All target data were obtained from NOX cultured L. gasseri WGS depths. We could not estimate the parameters of the Jones-Pewsey distribution based-model when a memory of less than 512 GB was used at a stride length of 1 nt. The figures on the right represent the required CPU time and maximum memory usage with varying coverage depth. The median filter used a window and stride length of 100 nt. All computations were performed via MAP estimation. [file peerj-08-8722-s006.pdf]

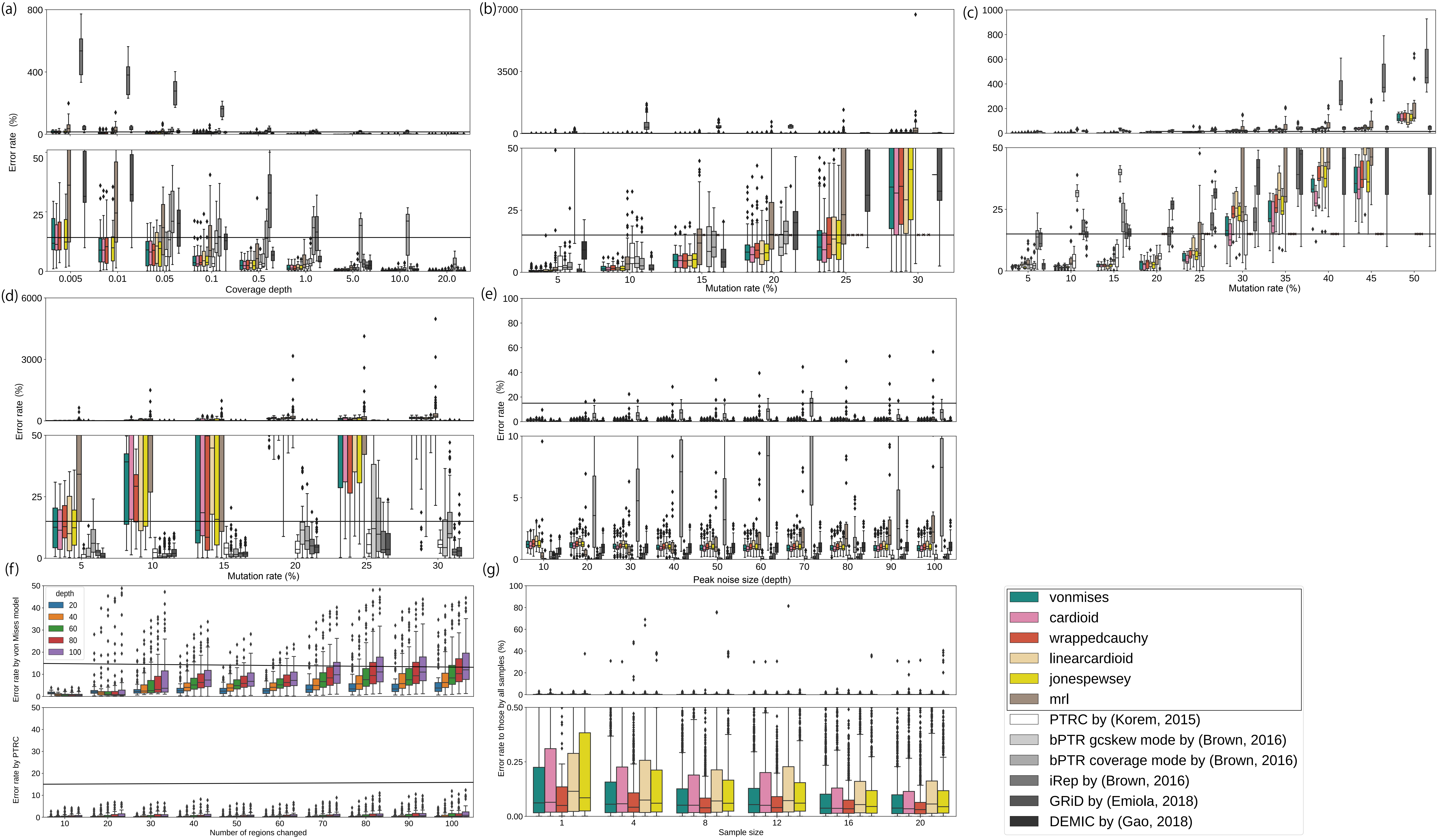

Supplement: Figure S7 — The robustness of the estimates was evaluated using artificial datasets. (A) The error rate for each average coverage depth was calculated with respect to the full coverage depth. Only the L. gasseri WGSs that had greater than 20 × coverage were used. The reference genome sequence was modified by (B) nucleotide level mutation, (C) block-level mutation on a random position, or (D) block-level mutation on a single specific region. The WGS was contaminated by artificial reads from the reference genome sequence at (E) a single position or (F) multiple positions so that the peak depth could be generated. (G) Even when we decreased the sample size, the effect was not substantial compared to the full set of samples. The horizontal bar represents the 15% threshold of the error rate defined previously (Brown et al., 2016). The black crosses on the bar indicate that the method was unavailable for mutated genome sequences. The WGS datasets were obtained from previous studies (Korem et al., 2015; Franzosa et al., 2018). The proposed models and statistics are placed in the black rectangle within the legend. [file peerj-08-8722-s007.pdf]

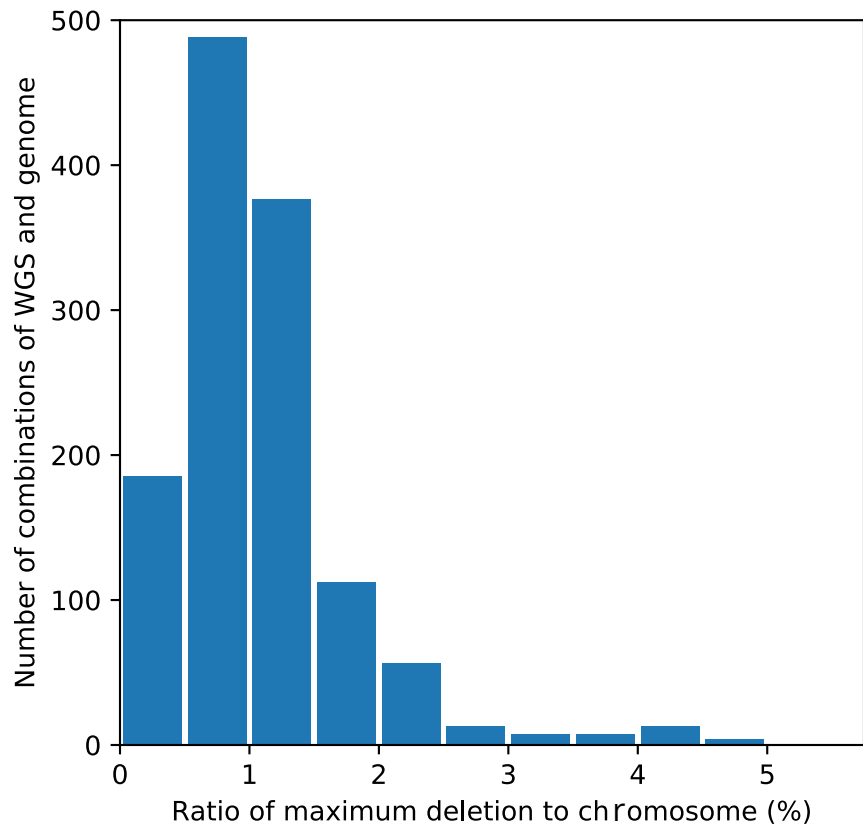

Supplement: Figure S8 — The size of the largest deletion was estimated from the coverage depth obtained when mapping the fecal metagenomic sequence of a human IBD patient cohort study (Franzosa et al., 2018) to a complete genome sequence database. [file peerj-08-8722-s008.pdf]

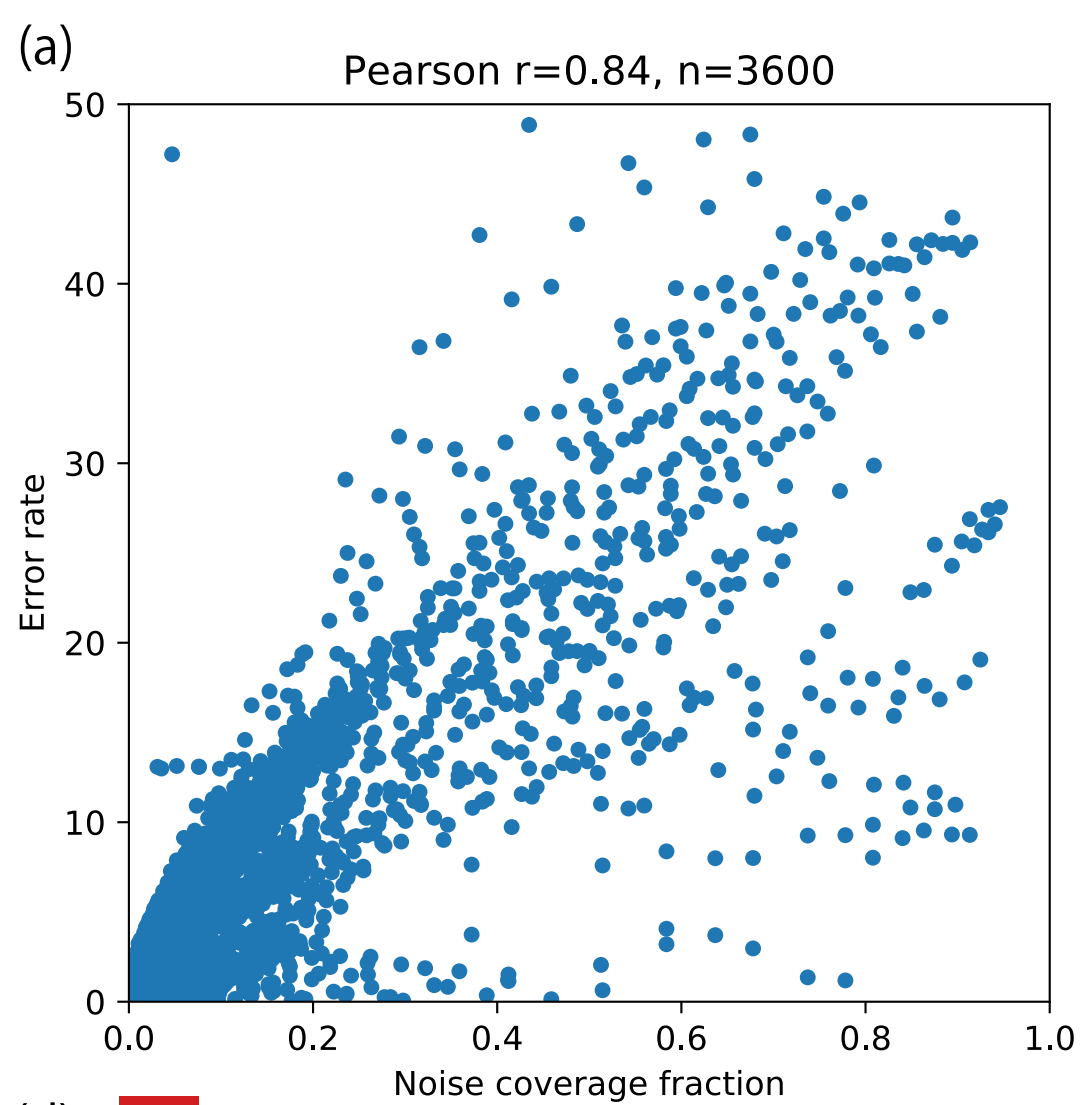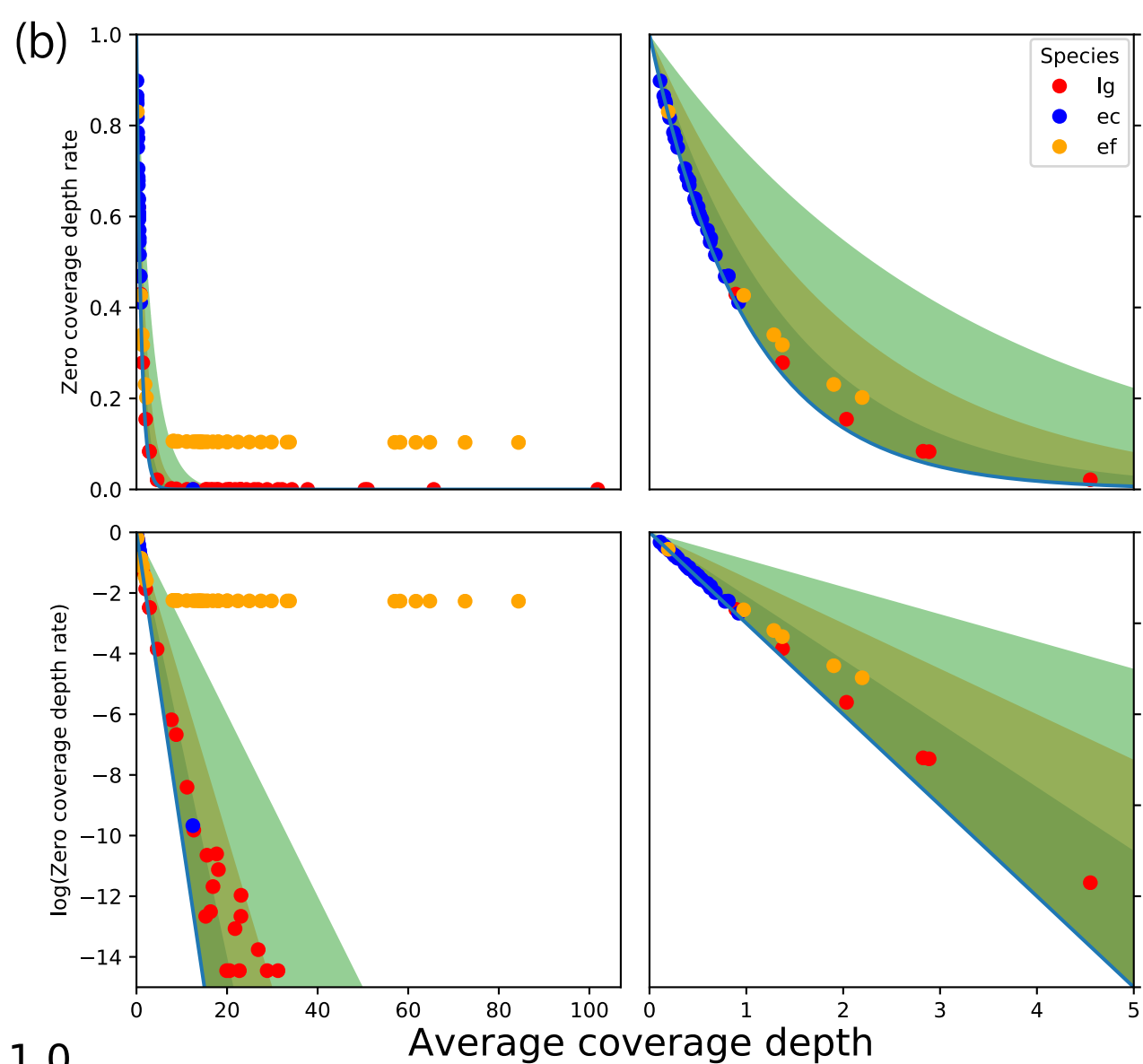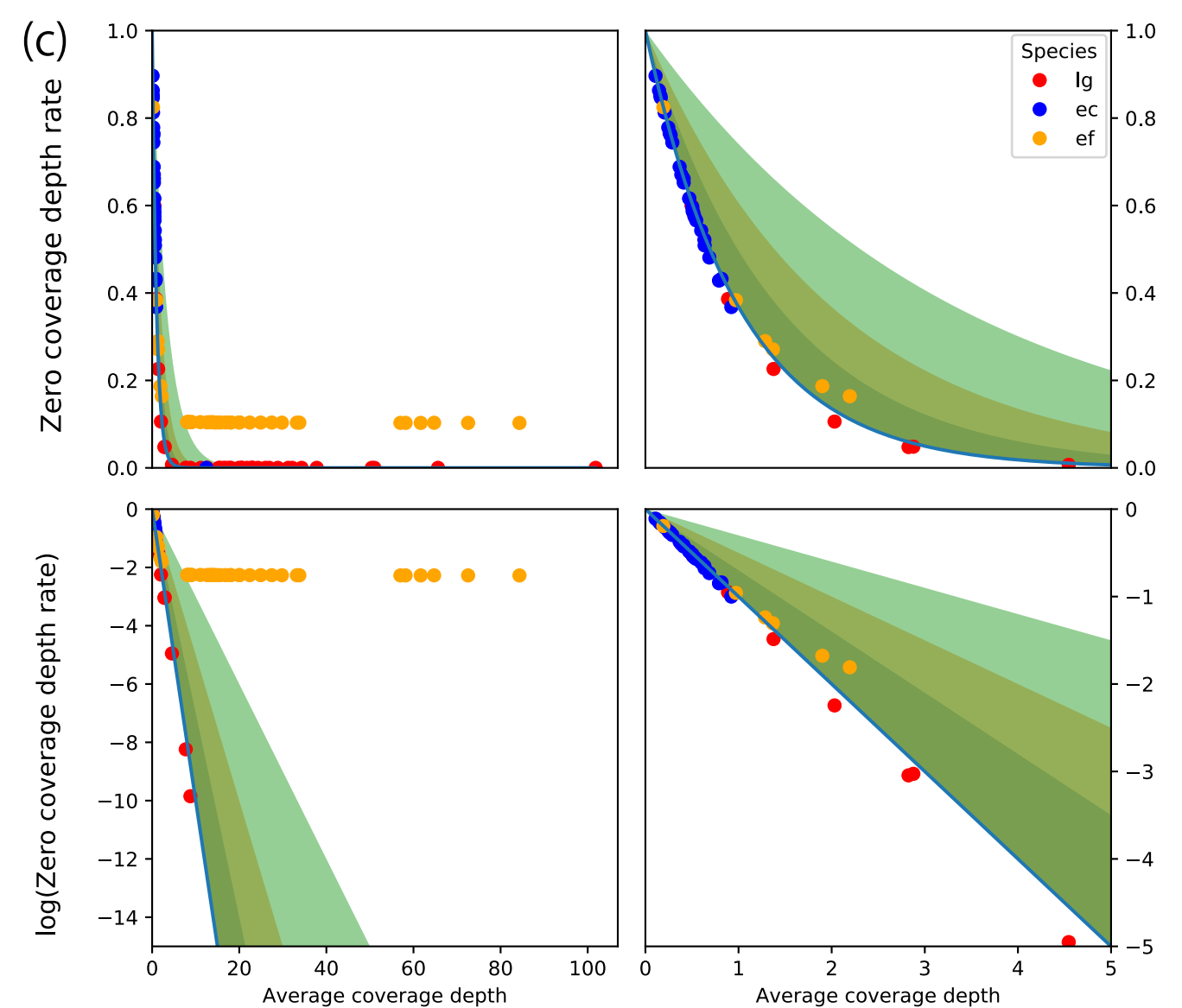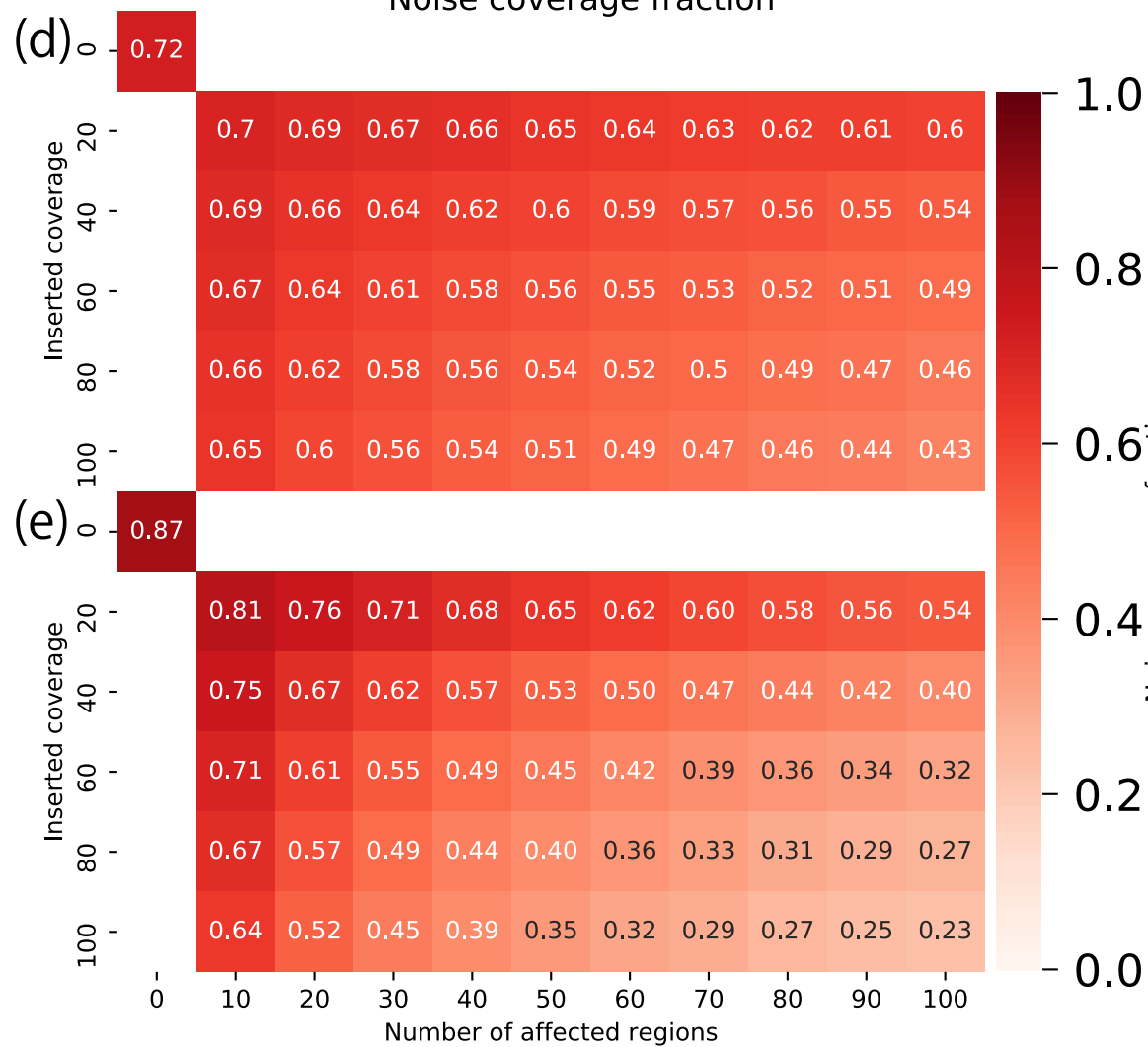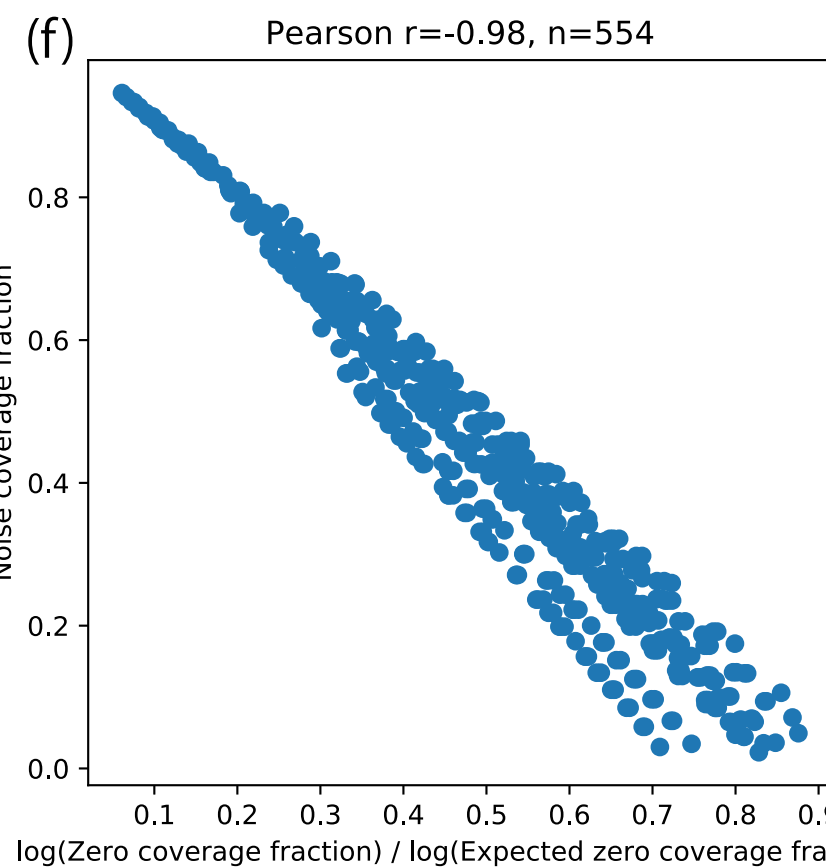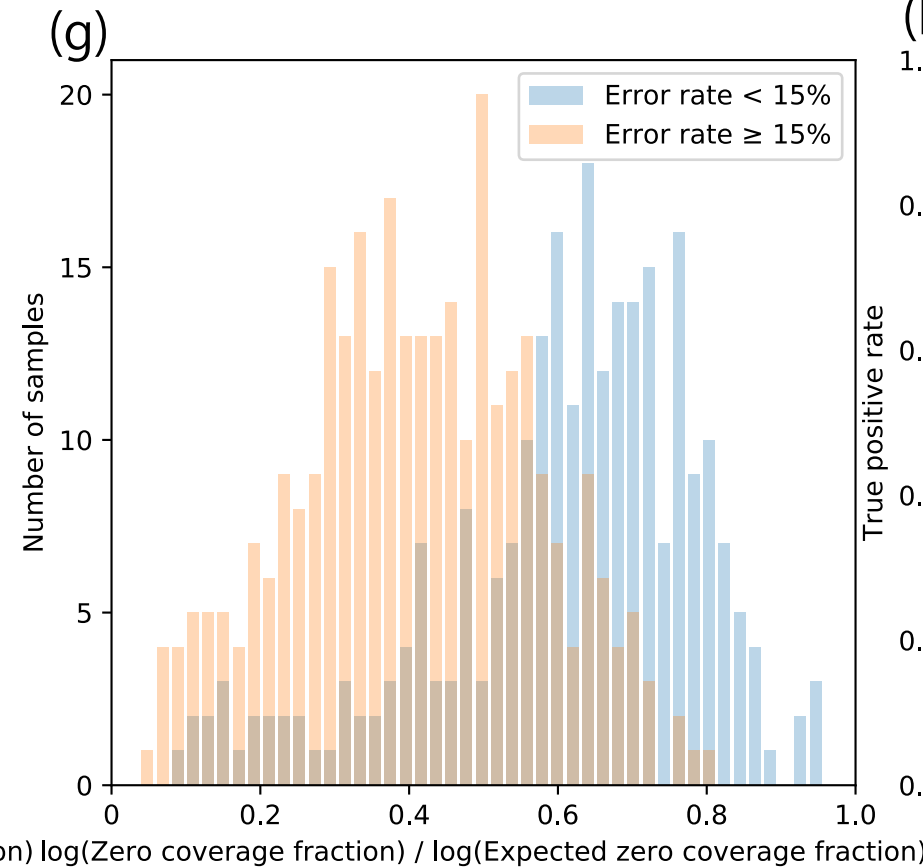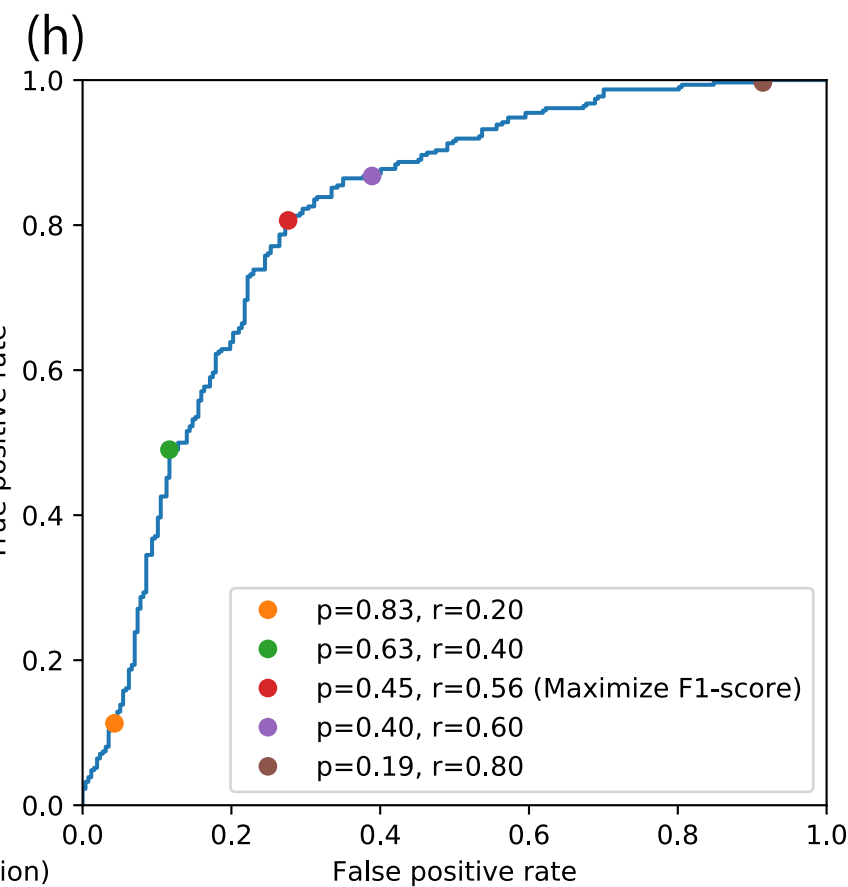

Supplement: Figure S9 — To infer the noise fraction in the coverage depth, we investigated the relationship with the zero-coverage depth fraction. (A) Correlation of the noise fraction with the error of our estimates in samples with more than 5.0 × average coverage. The dataset with multiple artificial peaked noise coverage was used. Evaluation of the Lander-Waterman theory in E. coli, E. faecalis, and L. gasseri datasets for (B) raw coverage depth datasets and (C) datasets after applying the moving median filter. We used a theoretical model assuming uniform probability. The variation ranges were visualized by 30%, 50%, and 70% of the log-scale score, respectively. Mean fold change between the log zero coverage fraction and theoretical score using (D) all noise-contaminated samples and (E) samples with less than 5.0 × average coverage. (F) Correlation between the noise coverage fraction and the fold change of the log zero coverage fraction in samples with less than 5.0 × average coverage. (G) Sample distribution with respect to the log zero coverage fraction. (H) ROC curve for estimation error determination when the threshold of the log zero coverage fraction was changed (AUC: 0.81). At the threshold for maximizing the F1 score, the accuracy was 0.77, the precision 0.78, and the recall 0.81. [file peerj-08-8722-s009.pdf]

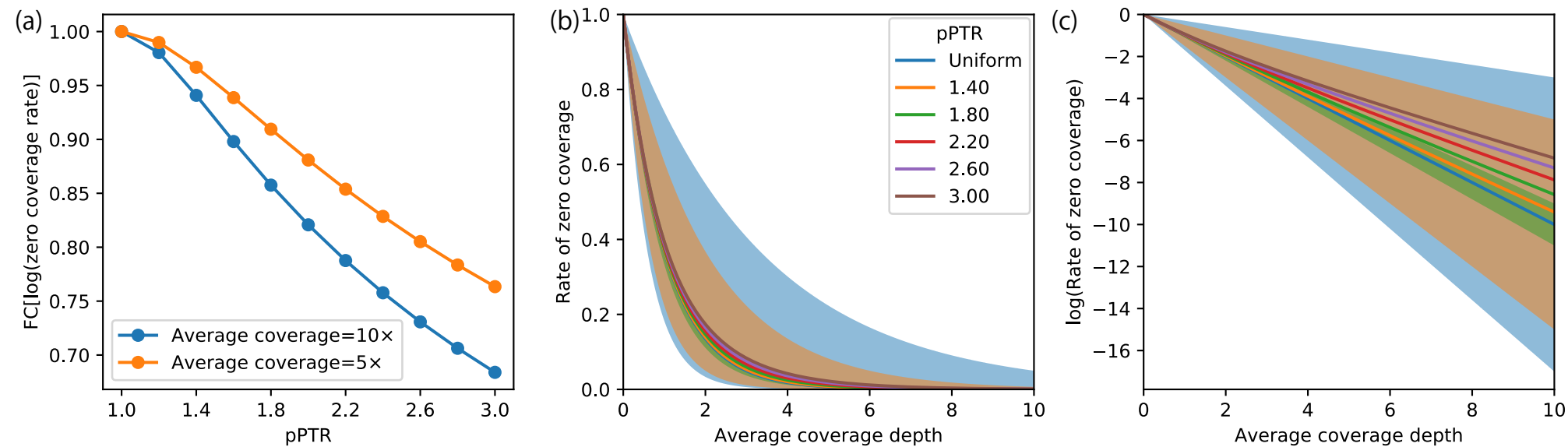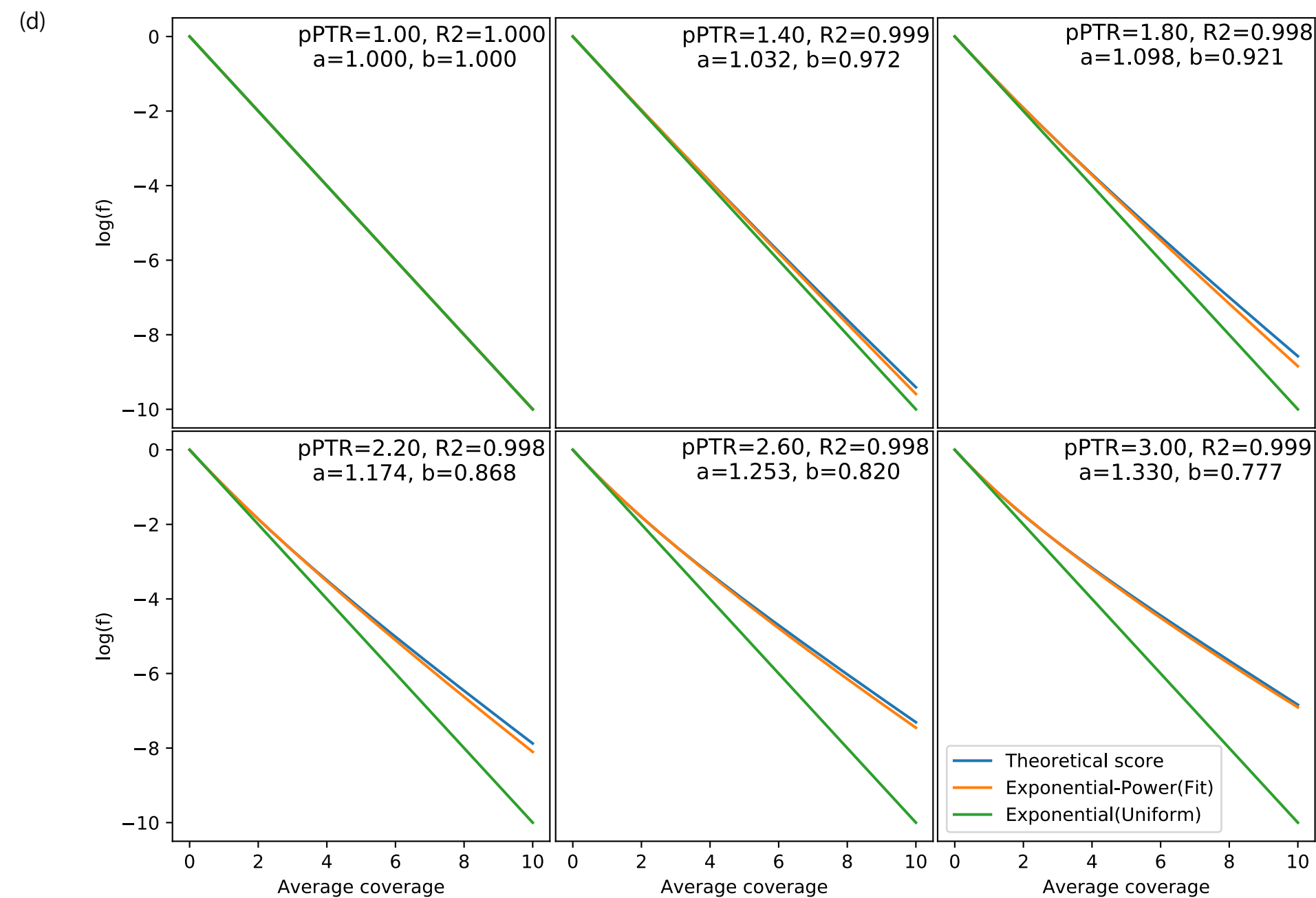

Supplement: Figure S10 — Based on the theory of Lander and Waterman, we investigated the relationship between the zero-coverage fraction and average coverage. We set the simulated sequence size to 10,000 nt. The observation probability at each nucleotide position was determined based on the von Mises distribution. (A) The y-axis represents the fold change of the log-transformed zero coverage fraction to that assuming uniform probability. The greater the PTR, the greater the deviation from the model assuming uniformity. This difference increases particularly when the average coverage is large. (B) Theoretical zero coverage fraction based on the Lander-Waterman theory when the pPTR was changed. The variation ranges in a model assuming uniform probability are shown at 30%, 50%, and 70% of the log-scale scores. (C) Log-scale score. (D) Fitting of the exponential-power function to the theoretical scores. The parameter of the function was determined via the least-squares method using Scipy. [file peerj-08-8722-s010.pdf]

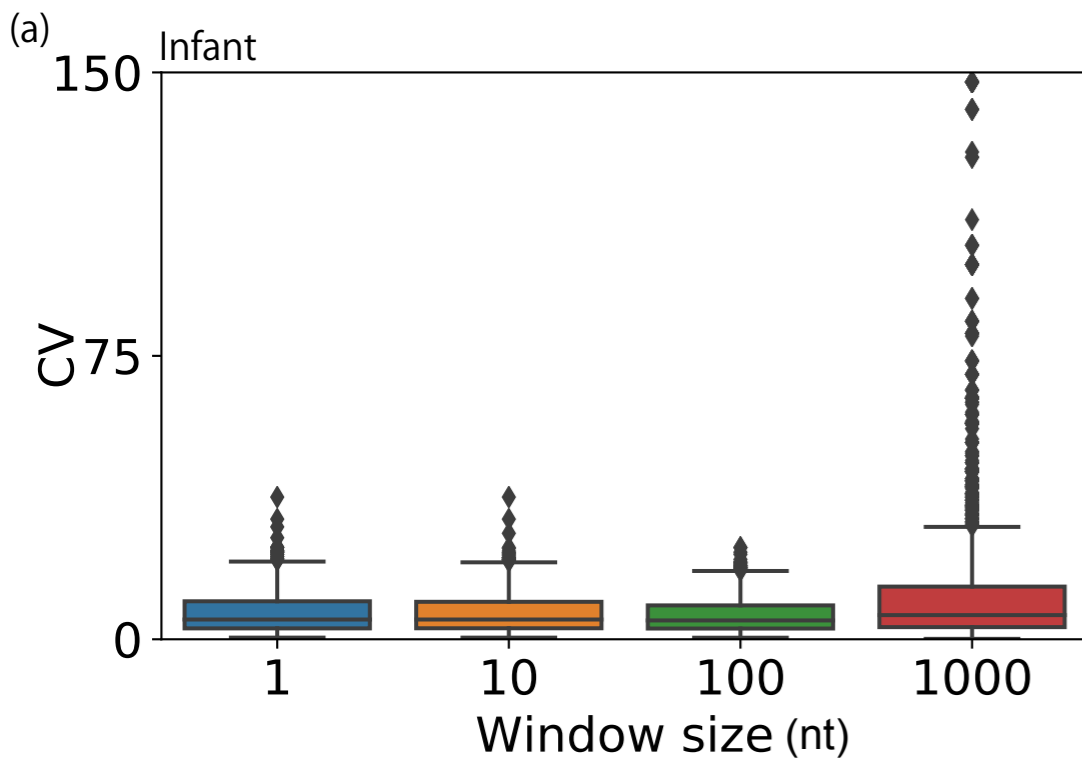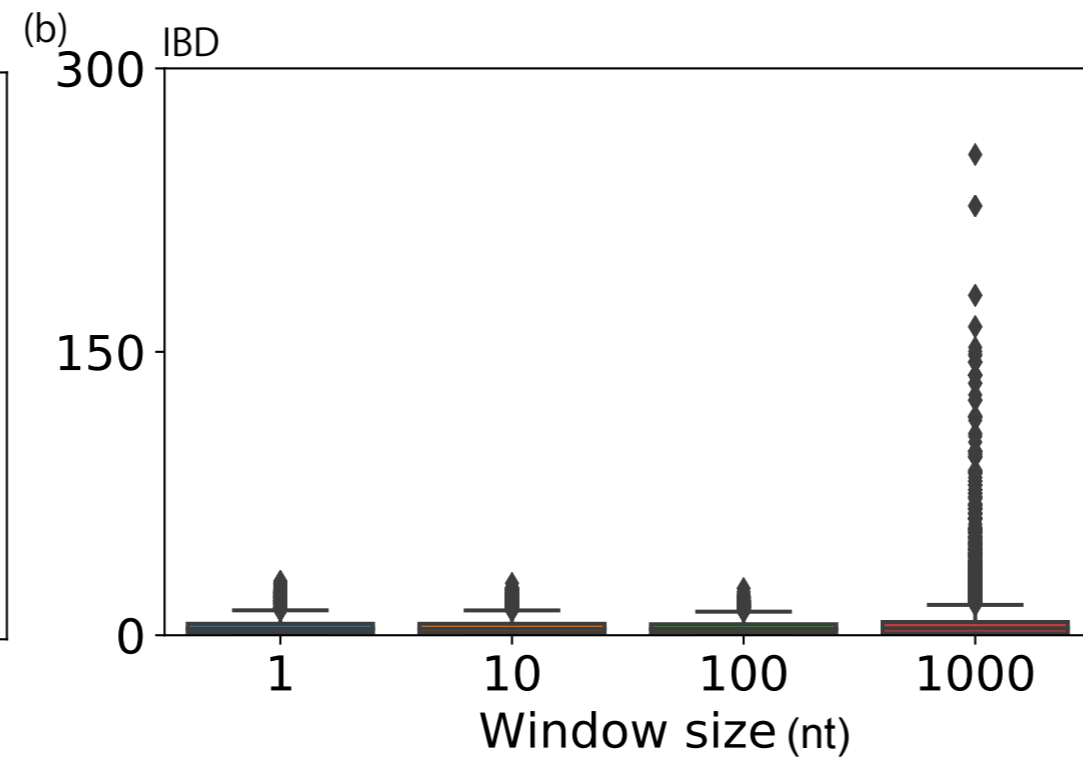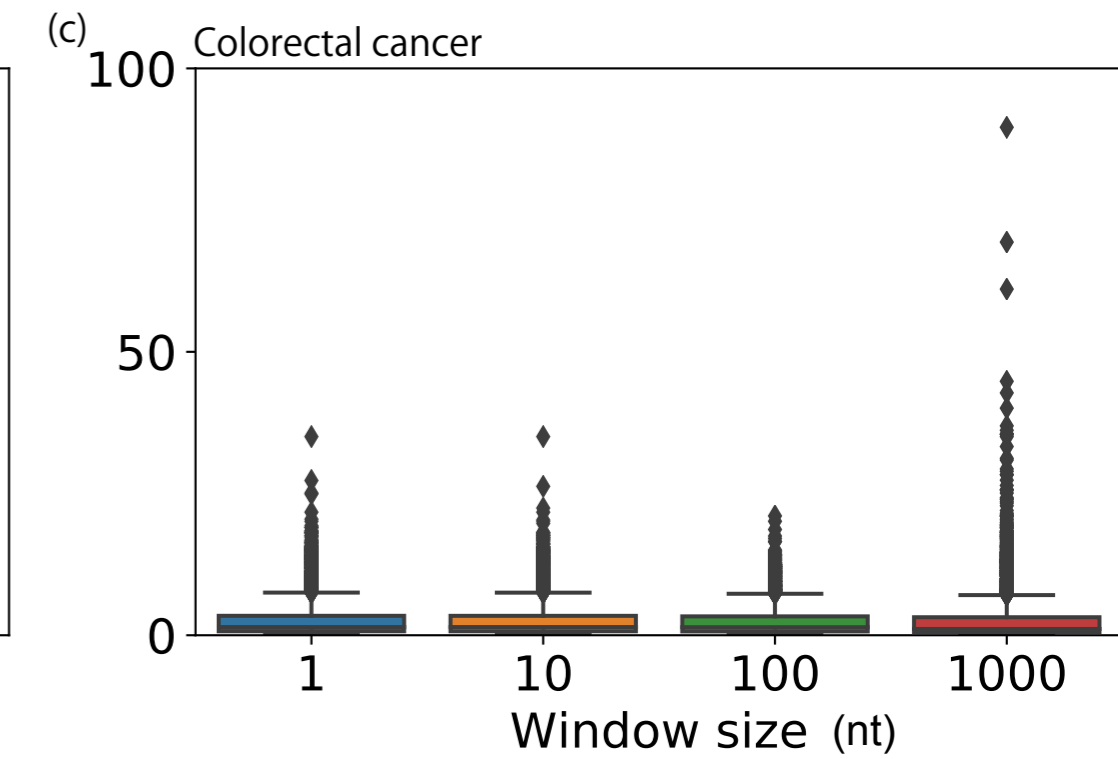

Supplement: Figure S11 — In the real metagenomic dataset, noise smoothing of the coverage depth by the median filter was evaluated based on the coefficient of variation. The data were obtained from human intestinal metagenomic sequences related to (A) infants (Bäckhed, et al., 2015), (B) IBD (Franzosa et al., 2018), and (C) colorectal cancer (Yu, et al., 2015). [file peerj-08-8722-s011.pdf]

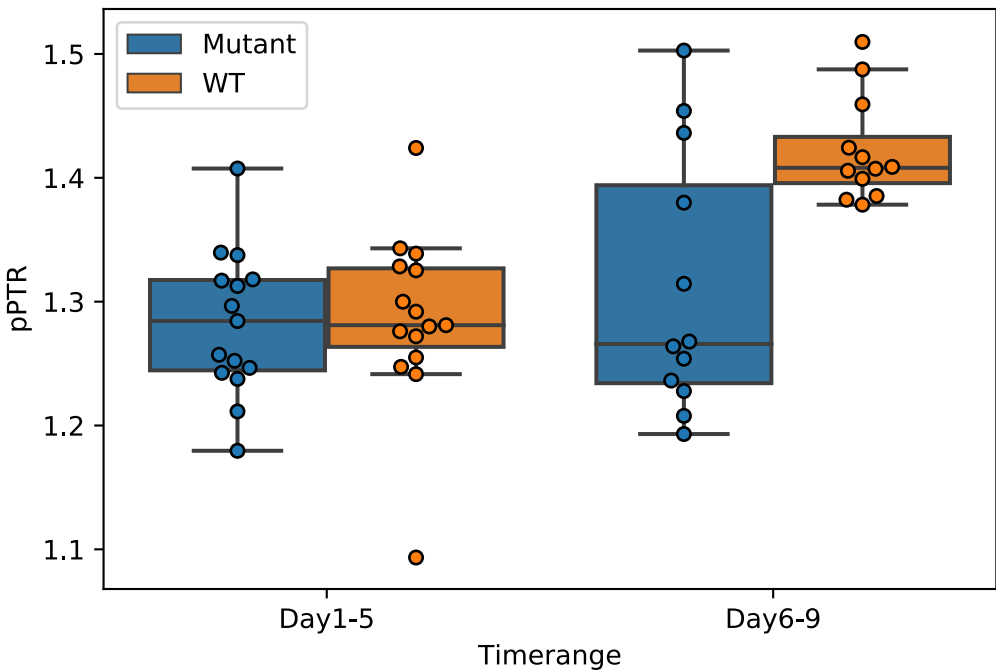

Supplement: Figure S12 — pPTRs were estimated using the von Mises distribution-based model. As in the work by Korem et al., significant differences were detected between the wild type and mutants on days 6–9 (FDR-corrected p-value: 8.72 ×10^−5^) and between days 1–5 and 6–9 for the wild type (FDR-corrected p-value: 1.04 × 10^−5^). [file peerj-08-8722-s012.pdf]

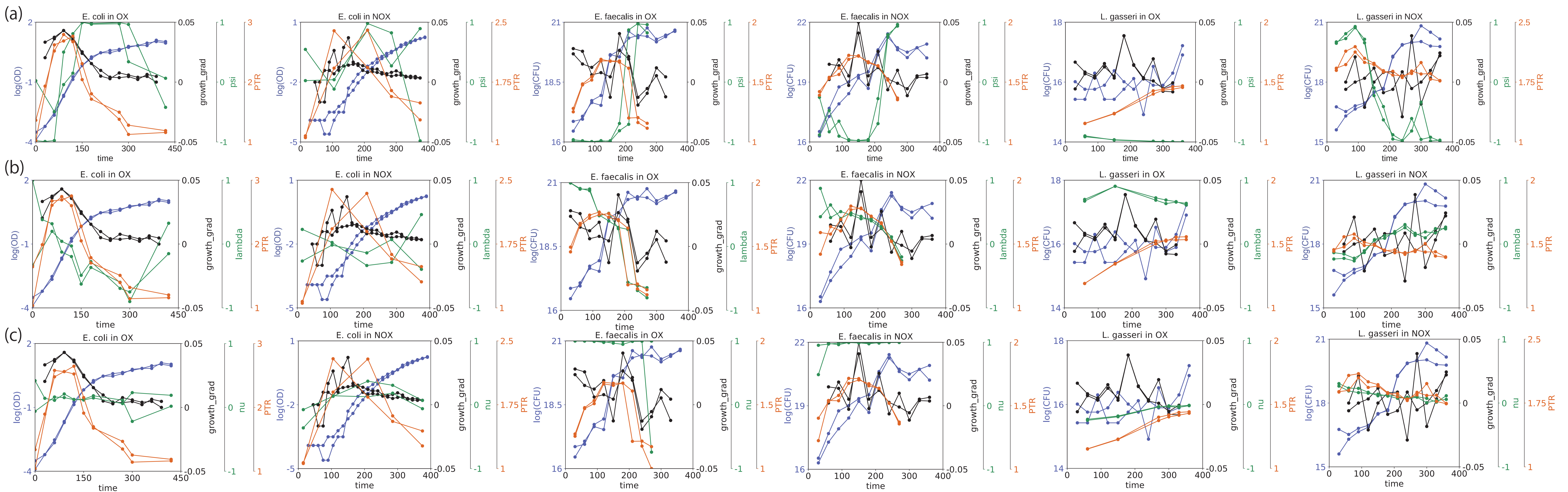

Supplement: Figure S14 — Degrees of density, peakedness, and skewness of replication according to the (A) Jones-Pewsey, (B) InvSE von Mises, and (C) InvMIAE von Mises distribution-based models. Except for the Jones-Pewsey distribution model for E. faecalis and L. gasseri, the parameters were estimated using the MAP estimation algorithm, while those of the other models were estimated using the MCMC algorithm. The estimates for all metagenomic samples obtained from the same species were obtained by sharing the location parameters regardless of the cultural state. The WGS datasets were obtained from Korem et al. [file peerj-08-8722-s014.pdf]

Pearson  $r=-0.793$ ( $p\_value=2.93e-07$ )

*E. coli*

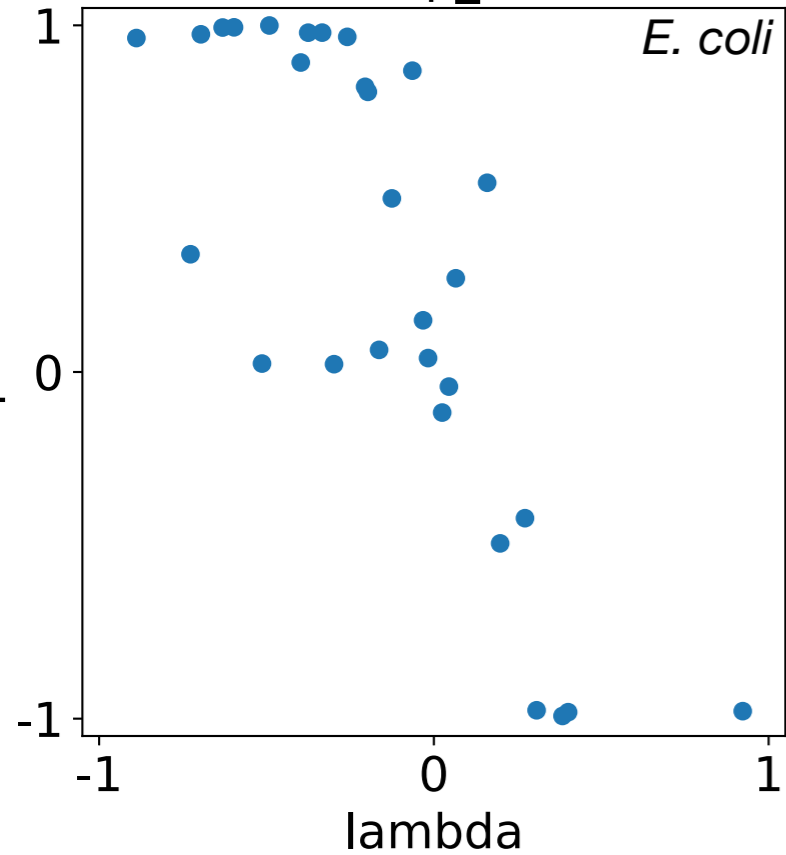

Pearson  $r=-0.878$ ( $p\_value=2.07e-12$ )

*E. faecalis*

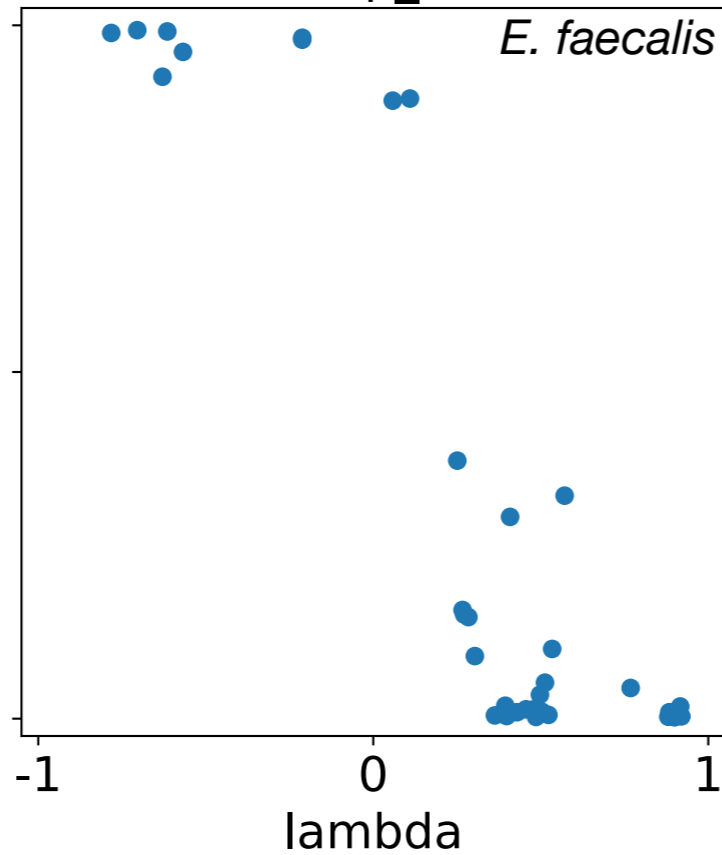

Pearson  $r=-0.809$ ( $p\_value=2.31e-09$ )

*L. gasseri*

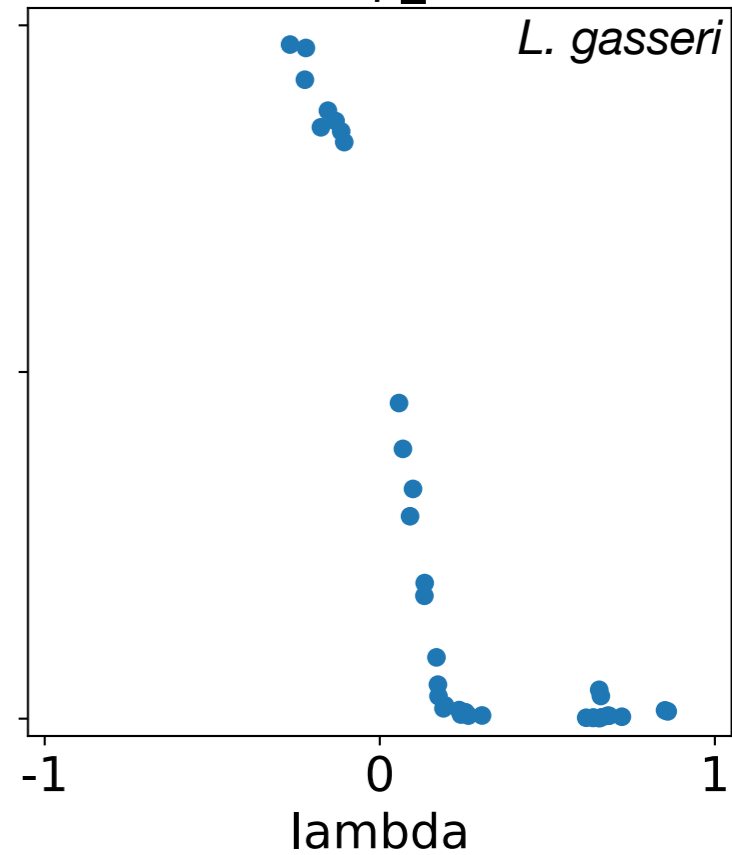

Supplement: Figure S15 — Scatter plot of the degree of concentration (psi) and peakedness (lambda). We calculated psi for E. coli and lambda using the MAP estimation, and calculated psi for E. faecalis and L. gasseri using the MCMC algorithm. The parameters were analyzed regardless of cultural status. [file peerj-08-8722-s015.pdf]

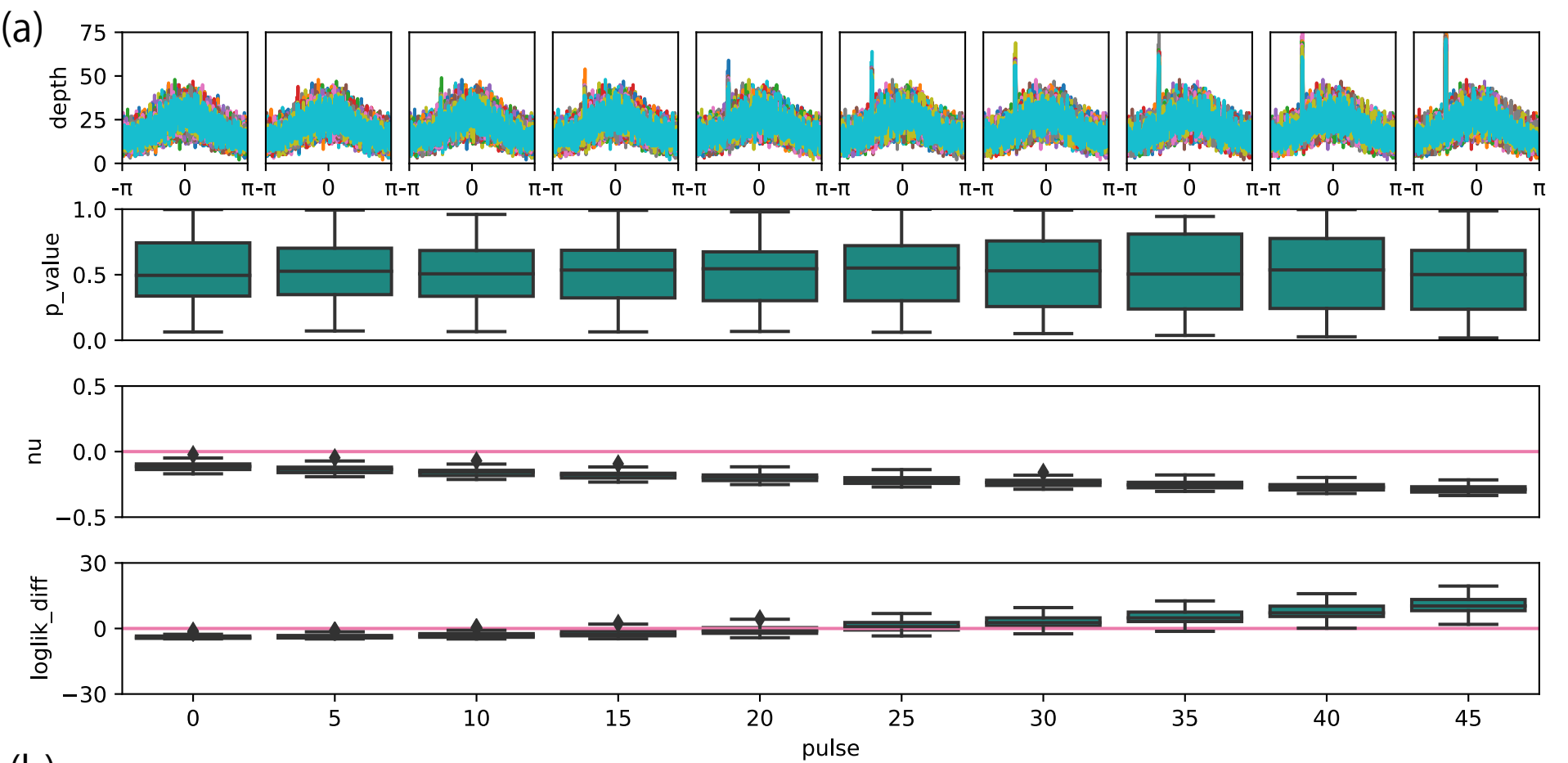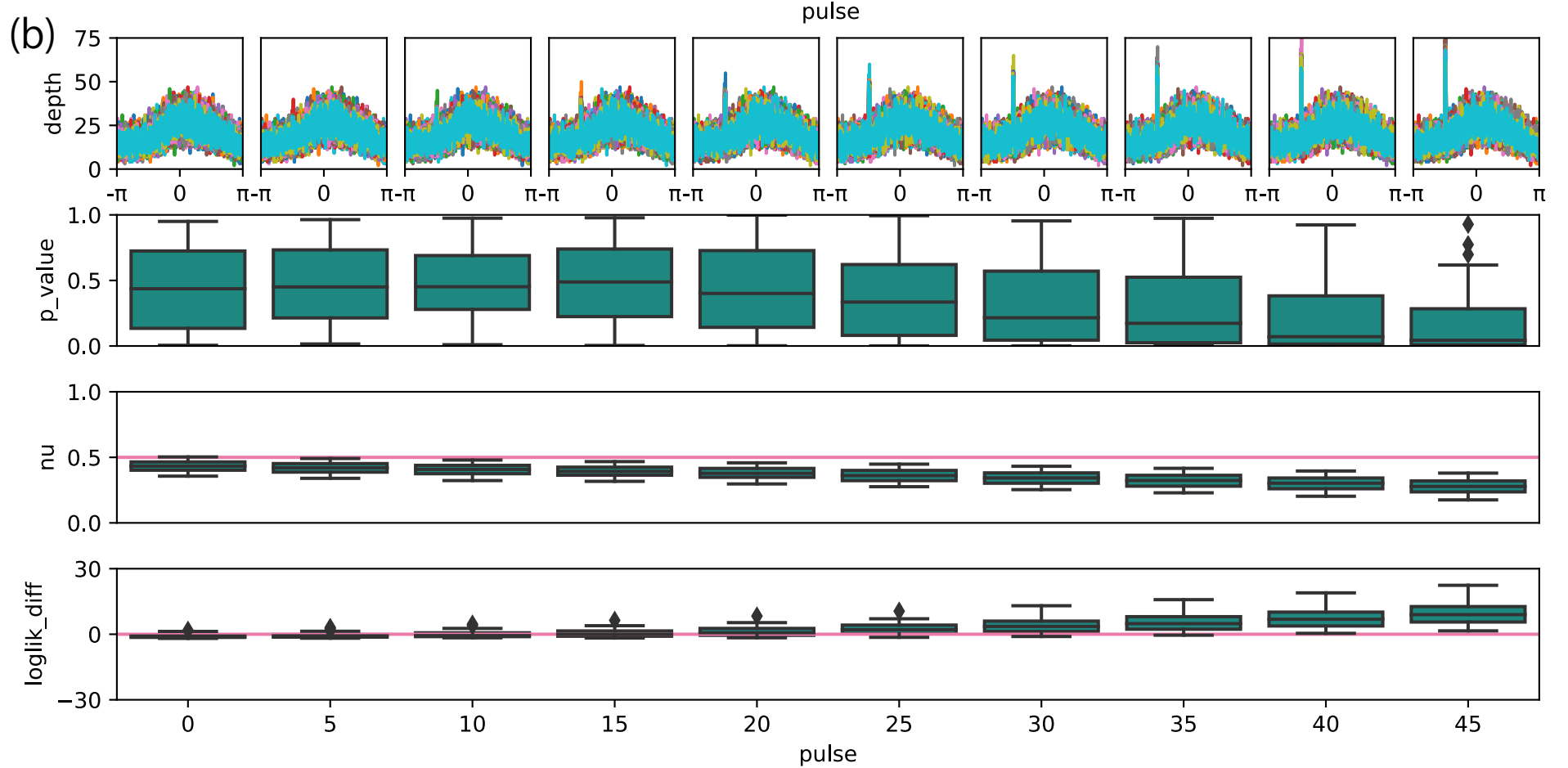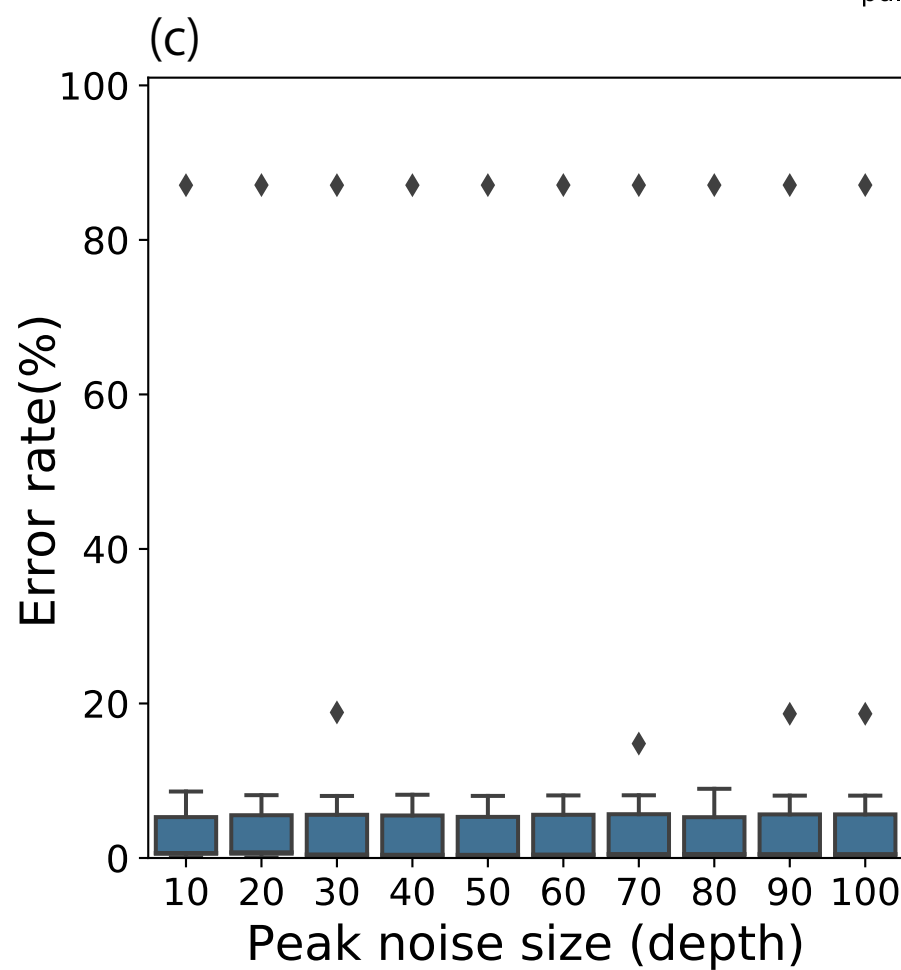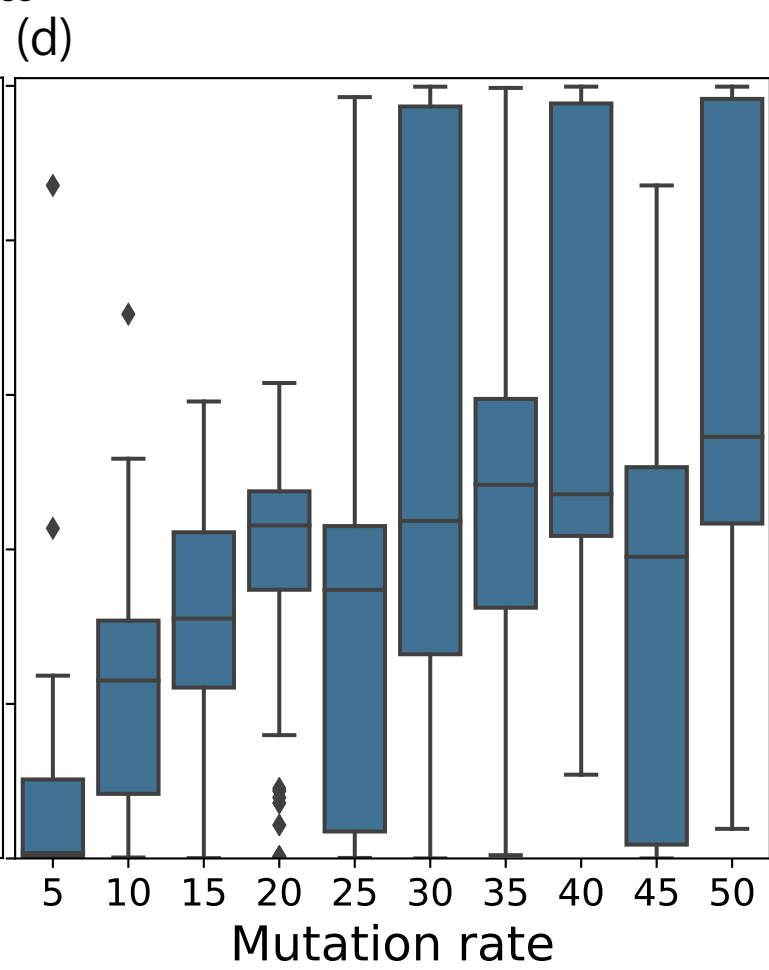

Supplement: Figure S16 — To evaluate the detection power in Pewsey’s asymmetry test and the invMIAE model, we computed the p-values of the test and skewness parameter of the model using the simulated dataset. Random numbers were iteratively generated following the (A) symmetric von Mises (nu = 0), and (B) asymmetric von Mises distribution (nu = 0.5) and multinomial distributions 50 times for each pulse strength. Pulse noise was added to the position that nearest to a quarter of the unit circle. The log-likelihood difference between the pulse-less true model and the estimated model is shown in the bottom row in each figure. The robustness of the model was also evaluated using (C) artificial peak noise and (D) an artificial mutation dataset. [file peerj-08-8722-s016.pdf]

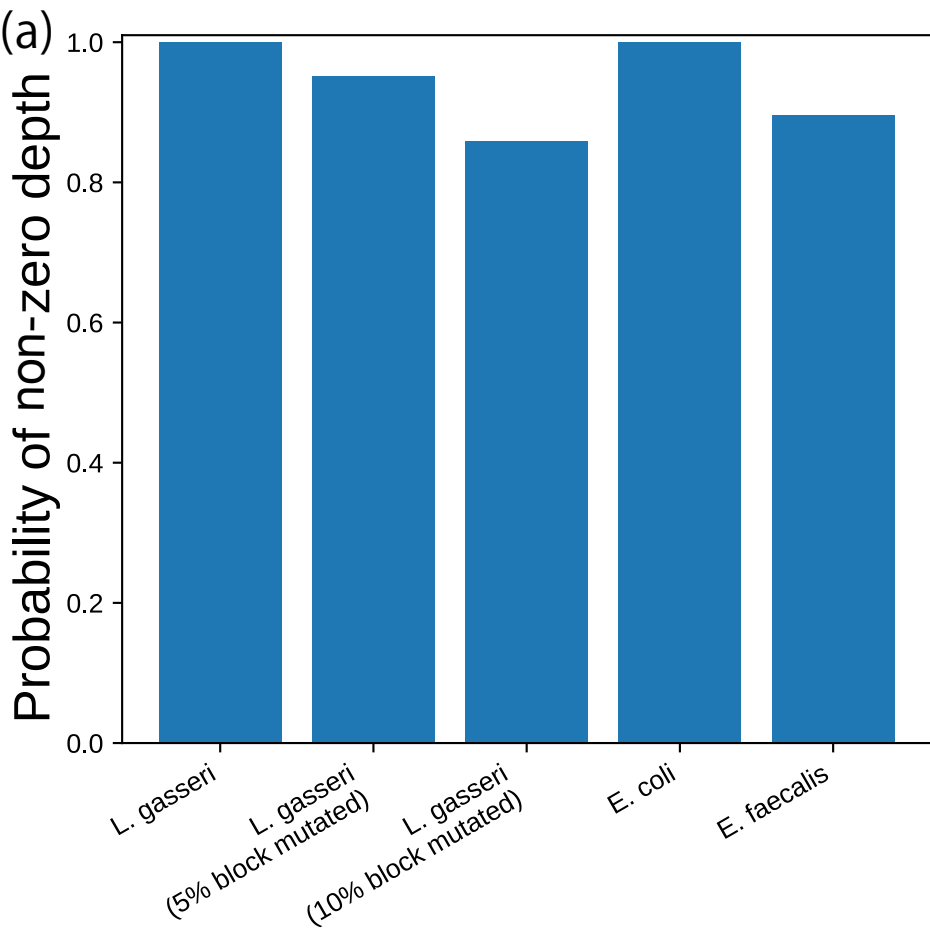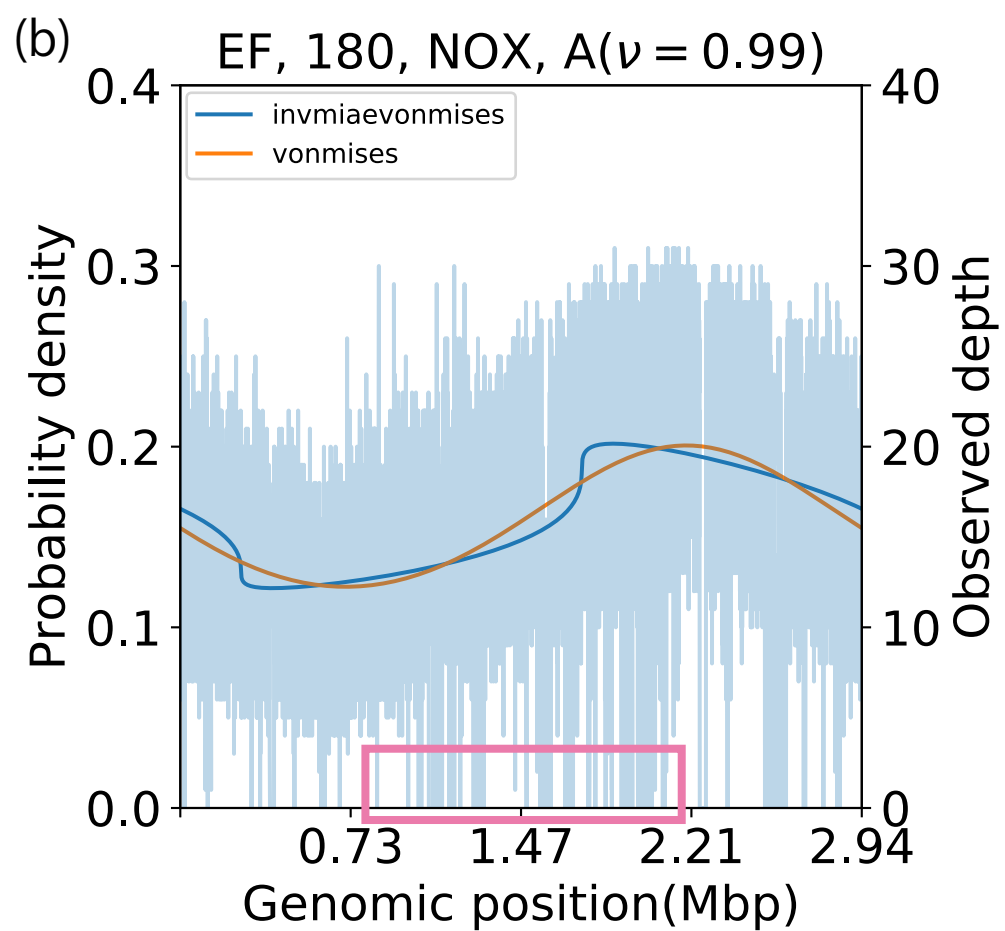

Supplement: Figure S17 — The pink square indicates the suspectable region that made the model skew. [file peerj-08-8722-s017.pdf]

Pearson  $r=0.996$ ,  $p\text{-value}=1.649\text{e-}105$

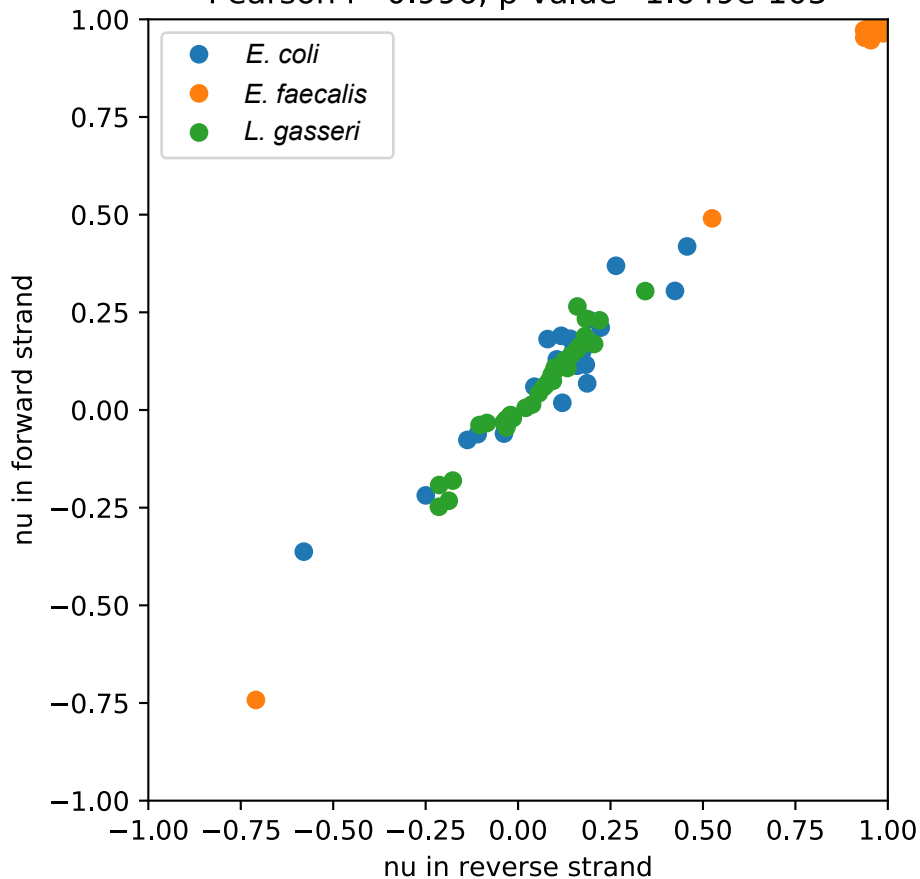

Supplement: Figure S18 — The parameters for each species were estimated by performing separate optimization trials. The correlation coefficient and p-value were calculated using all measurements. [file peerj-08-8722-s018.pdf]

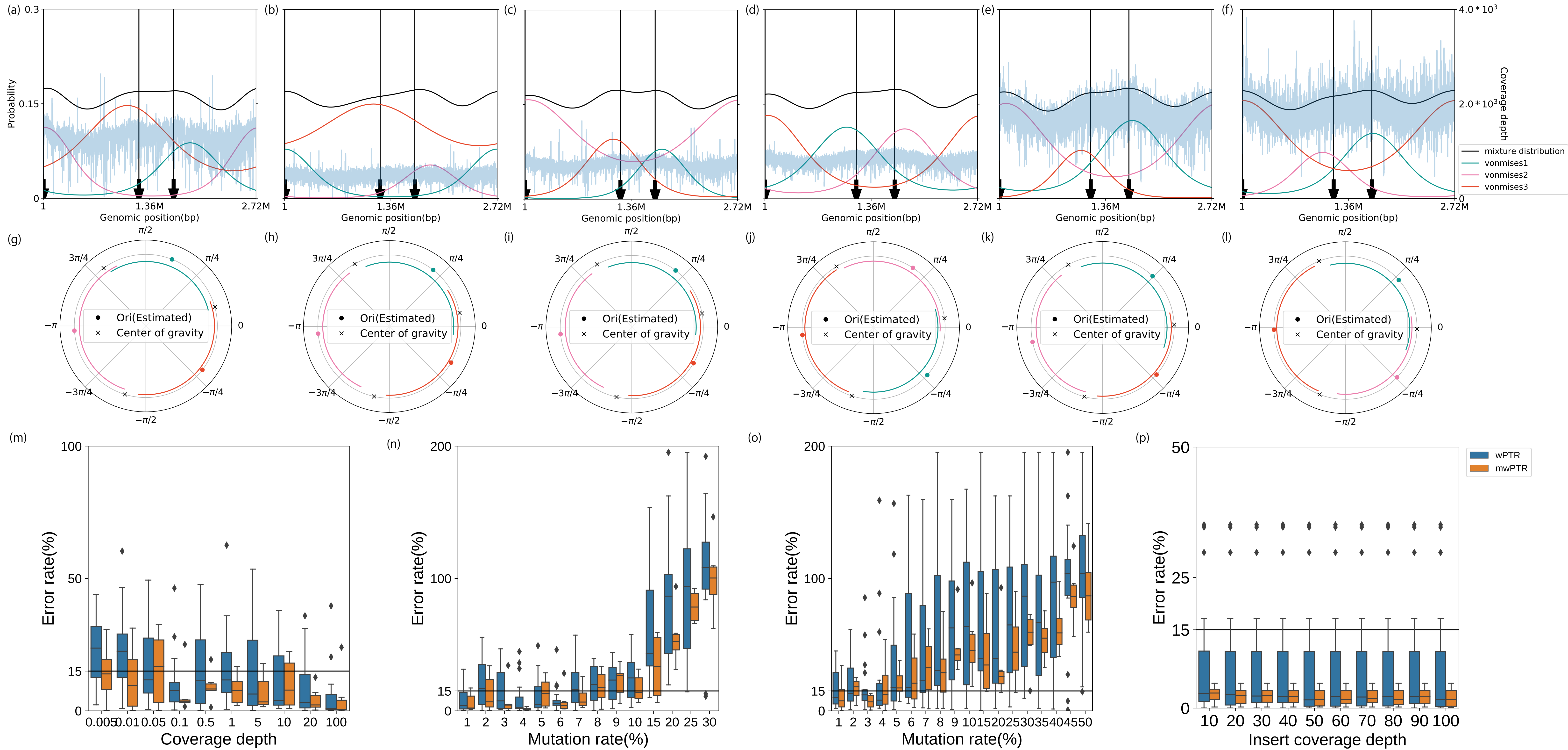

Supplement: Figure S19 — Growth rate estimates were performed on S. solfataricus using data sets obtained from Payne, et al. Subplots depict datasets from (A, G) SARC-B, (B, H) SARC-C, (C, I) SULA, (D, J) SULG, (E, K) SARC-H, and (F, L) SARC-I. Robustness of the wPTR and mwPTR estimates was evaluated using artificial datasets with (M) decreased coverage depth, (N) point mutation applied, (O) 5,000 nt block mutation applied, and (P) peaked noise inserted. [file peerj-08-8722-s019.pdf]
