# Supplementary material for: Probabilistic model based on circular statistics for quantifying coverage depth dynamics originating from DNA replication
Source: PeerJ. 2020 Mar 27;8:e8722. doi: 10.7717/peerj.8722 (PMC7104724; doi:10.7717/peerj.8722)
Supplement: Figure S13 — Estimated growth of B. adolescentis and B. breve in fecal samples from neonates and their mothers obtained from the (A) von Mises distribution model and (B) PTRC. This cohort was composed of 100 neonates and their mothers. Three fecal samples were obtained from the neonates just after birth, four months after birth, and one year after birth. The feces of the mothers were sampled just after birth. Bars with single stars indicate significant differences in the growth estimates according to Welch’s t-test (p-value ≤ 0.05), and the bars with double stars indicate significant differences after FDR correction (FDR-corrected p-value ≤0.05). For the correction, we followed the Benjamini-Hochberg procedure. [file peerj-08-8722-s013.pdf]

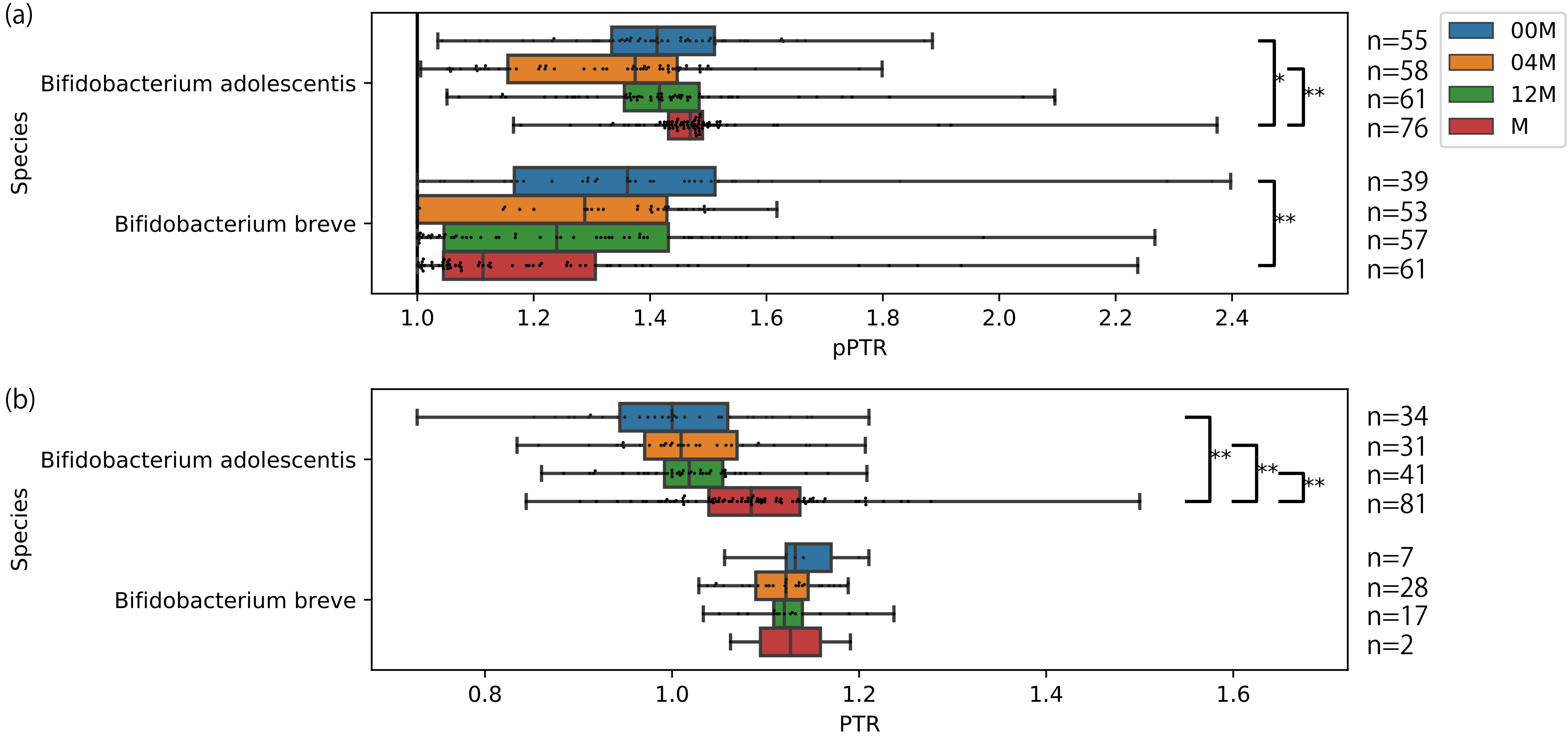

(a)
Bifidobacterium adolescentis
Bifidobacterium breve
Species
pPTR
1.0
1.2
1.4
1.6
1.8
2.0
2.2
2.4
n=55
n=58
n=61
n=76
n=39
n=53
n=57
n=61
*
**
**
00M
04M
12M
M
(b)
Bifidobacterium adolescentis
Bifidobacterium breve
Species
PTR
0.8
1.0
1.2
1.4
1.6
n=34
n=31
n=41
n=81
n=7
n=28
n=17
n=2
**
**
**
