# Supplementary material for: Probabilistic model based on circular statistics for quantifying coverage depth dynamics originating from DNA replication
Source: PeerJ. 2020 Mar 27;8:e8722. doi: 10.7717/peerj.8722 (PMC7104724; doi:10.7717/peerj.8722)
Supplement: Supplemental Information 1 [file peerj-08-8722-s030.pdf]

Supplement Materials
Contents

Supplementary texts 2

1 Evaluation of estimation ability in discrete coverage depth by continuous distribution 2

2 Relationship between peak-trough ratio of coverage depth and probability 2

3 Determination of filtering threshold for peak noise elimination 3

4 Estimation of noise fraction in coverage depth 4

5 Creation of artificial coverage depth 5

6 Effect of moving median filter parameter on the correlation between the growth estimates and experimental growth rate 5

7 Effect of coverage depth decrease at the edge of the genome 6

8 Distribution of the maximum continuous zero coverage size in the IBD dataset ... 7

9 Approximation of zero coverage fraction with replication effects 7

10 Simulation dataset to evaluate the performance of skewness detection 8

11 Determination of applicable threshold for asymmetric extended model 8

Supplementary texts

1 *Evaluation of estimation ability in discrete coverage depth by continuous distribution*

If the angle is considered discrete, either the normalization constraint c_{ω} should be adjusted such that $P_{discrete}(\theta \mid \omega) = \frac{1}{c_{\omega}} P_{continuous}(\theta \mid \omega)$ and $c_{\omega} = \sum_{i=1}^{n} P_{continuous}(\theta_i \mid \omega)$ or the probability mass function of the truncated depths should be calculated such that $P_{discrete}(\theta \mid \omega) = \int_{\theta-\delta}^{\theta+\delta} P_{continuous}(\theta \mid \omega) d\theta$, where δ is a minute interval of the angle satisfying $\delta = \frac{2\pi}{I-2}$. However, these procedures require extensive computational resources, as *n* matches the size of the input sequence. Therefore, continuous distribution was directly employed to reduce the use of resources required for estimation. We evaluated the possible error which this procedure may cause in the model by fitting the model to a simulation dataset (Supplementary Fig. 2; see *Methods* for the procedure). As the error rates of our model were similar to those of the normalized model, we concluded that this procedure did not affect the final results.

2 *Relationship between peak-trough ratio of coverage depth and probability*

Transformation of the coverage depth into probability by expectation implies that only considering the ratio of maximum to minimum depth does not yield a properly estimate of the bias, as follows:

$$\frac{\mathrm{Exp}(d)_{\max}}{\mathrm{Exp}(d)_{\min}} = \frac{\mathrm{Exp}\left(\mathrm{Binomial}\left(T_s, P\left(\theta = \frac{i_{\max}}{I} 2\pi | \omega\right)\right)\right)}{\mathrm{Exp}\left(\mathrm{Binomial}\left(T_s, P\left(\theta = \frac{i_{\min}}{I} 2\pi | \omega\right)\right)\right)}$$

$$= \frac{T_s P\left(\theta = \frac{i_{\max}}{I} 2\pi | \omega\right)}{T_s P\left(\theta = \frac{i_{\min}}{I} 2\pi | \omega\right)}$$

$$= \frac{p_{\max}(\theta)}{p_{\min}(\theta)}$$

$$\neq \frac{d_{\max}}{d_{\min}}$$

where $i_{\max}$ and $i_{\min}$ are defined as the positions that assume maximum or minimum probability, respectively. Due to observation errors represented by the variance of the distribution $\mathrm{Var}(d_i) = T_s p_i (1 - p_i)$, the position with the maximum raw coverage depth cannot be the position with the highest probability. However, an accurate estimate could be achieved by reducing this variance, as was done using the moving median filter in previous studies.

3 Determination of filtering threshold for peak noise elimination

The threshold employed to remove peak noise was determined using a statistical distribution. We constructed a model where the coverage depth d of the overall genome sequence followed a discrete probability distribution P with parameter set ξ (i.e., $d \sim P(\xi)$). This approach assumes that the peak noise is located as an outlier in the depth distribution throughout the WGS. When we compared the probability distributions for multiple data sets, the zero-inflated negative binomial distribution had the best score (Supplementary Table 5). We determined the top 1% to be the threshold based on the cumulative density function score as well as a previous study (Brown et al., 2016). We evaluated the validity of the procedure to remove the top 1% of the coverage depth. First, we evaluated the effect of removing data that did not contain noise coverage. For this purpose, we fitted the zero-inflated negative binomial distribution to a coverage depth of *L. gasseri* (ERR969426). We visually confirmed that this sample did not contain significant

noise. Next, we quantified the position of the actual top 1% of coverage depth in the fitted distribution. It was confirmed that the actual top 1% of coverage was located at 98.9% in the cumulative distribution function, which was equivalent to the expectation multiplied by 2.57 times the rooted variance. This result implies that the removal procedure affected a small portion of coverage on the noise-free distribution. Second, we verified the effect of removing noise. For this purpose, we constructed a coverage depth with noise by inserting 100 copies of part of the reference sequence (1,000 nt) into the sequence reads of *L. gasseri*. Next, four types of binned coverage were created by applying only the moving median filter or top 1% removal, followed by the moving median filter, to the original or noisy coverage, respectively. Next, a von Mises distribution was fitted to each dataset. Finally, the distance between the probability distributions was calculated. The results confirmed that the probability distribution obtained by applying the top 1% noise reduction was similar to the original distribution. It was also confirmed that the effect of removing the top 1% coverage from noiseless data was smaller than the noise itself. Thus, we concluded that removing the top 1% has a slight effect on the noise-free distribution and provides noise reduction.

4 *Estimation of noise fraction in coverage depth*

In the performance evaluation, we confirmed that a large amount of noise affects our statistical model based on a circular distribution. When we conducted the evaluation using the dataset, the error rate was positively correlated with the noise coverage fraction for all coverage depths (Pearson r = 0.84, n = 3,600); (Supplementary Fig. 10a). This finding indicates that the main reason for the worsening of the estimate was that the noise broke the random sampling premise. In an actual situation, the proportion of noise is unknown and must be estimated. The noise contamination is particularly important for low-coverage datasets, because it is difficult to visually detect noise for a low-coverage dataset. Additionally, a low-coverage dataset may be relatively sensitive to noise. For this purpose, we focused on a rate at which the coverage depth is 0. First, as this phenomenon was modeled in previous studies (Lander & Waterman, 1988; Roach, 1995), we confirmed the effect of replication on the model. As the PTR increased, the theoretical score deviated from the score assuming uniform probability, but the difference decreased when the average coverage depth was small (Supplementary Fig. 11). Second, when we verified the theoretical score in the less than 5.0× coverage dataset of *E. coli*, *E. faecalis*, and *L. gasseri* WGS reads (n = 101), the

zero-coverage fraction fitted the theoretical score with an error rate of 16.3% (Supplementary Figs. 10b and c; Supplementary Table 6). The samples with artificial coverage noise departed from the theory as the amount of the noise increased (Supplementary Figs. 10d and e). As we could confirm the correlation with the fold change of log-transformed zero coverage fraction with the theoretical model and the noise fraction (Supplementary Fig. 10f), we evaluated the performance to use it as a marker of noise contamination (Supplementary Figs. 10g and h). Resultingly, we confirmed that 81% of the invalid coverage depth distribution for our model could be detected when the threshold of the fold change was set at 0.56.

5 *Creation of artificial coverage depth*

To validate our model, we generated an artificial coverage depth from our model. We used von Mises, cardioid, wrapped Cauchy, Jones-Pewsey, and linear cardioid distributions as the circular distributions of the model. The length of the genome sequence was set to 1,000,000 nt. The location parameter of the circular distributions was set to be exactly in the middle of the discrete angle, and the concentration parameter was set such that the PTR became 2.0 (von Mises: 0.34657, cardioid: 0.16666, wrapped Cauchy: 0.17157, Jones-Pewsey: 0.34657, linear cardioid: 0.1061). The shape parameter of the Jones-Pewsey distribution was set to 0.5, so that the probability density function would be different from those of the von Mises distribution (which matches that of a Jones-Pewsey distribution when the shape parameter is 0), wrapped Cauchy distribution (1.0), and cardioid distribution (-1.0). Finally, we randomly sampled the angles with a multinomial distribution according to the probability density function of the circular distribution so that the average coverage depth became 20.0.

6 *Effect of moving median filter parameter on the correlation between the growth estimates and experimental growth rate*

We evaluated the effect of the moving median filter on the growth rate estimates by changing both the window size, which represents the range of each processed data point, and the stride length, which represents the interval of the window. We observed that the correlation between the estimates and experimental growth rate remained greater than 0.5 when both lengths were less than 100 bp (Supplementary Fig. 4). The purpose of the filter is to reduce the variance in the coverage depth and outliers. As such, if filtering is performed, ideally, the average

depth will be maintained, and the variance will decrease. We checked these statistics on the filtered data when both parameters were more than 100 nt and observed that the filter failed to maintain the average for a portion of the data. Resultingly, the ratio of the coefficient of variance decreased when the window length was less than or equal to 100 nt. However, it increased in samples from one *L. gasseri* NOX culture, one *E. faecalis* NOX culture, 12 *E. coli* OX cultures, and nine *E. coli* NOX cultures when the size was greater than 100 nt (Supplementary Fig. 5). We therefore concluded that the correlation coefficient between the growth rate estimates and the experimental growth rate is valid in cases with less than or equal to 100 nt of filtered data. When we evaluated the moving median filter with a large window size, the coefficient of variation of the coverage depth indicated that some samples did not reduce the variance, but rather increased it. This behavior is probably due to the filter converting the window depth to 0 when the coverage was low. In such cases, the shape of the original distribution was not preserved, preventing accurate estimation. Although a large window size is helpful for smoothing outliers, it necessitates checking the coverage depth statistics after filtering. Moreover, the smaller stride length of the filter probably provides better results as it passes large amounts of data for estimation; however, it requires large amounts of memory and computational time for estimation as the volume of data increases. Accordingly, this parameter should be changed depending on the expected precision.

7 *Effect of coverage depth decrease at the edge of the genome*

We evaluated the effects of the decrease in coverage depth observed at the edge of the genome. Ordinally, the FASTA file format stores template genome sequences in a linear form, and most sequence aligners will not convert the linear sequence into a circular form. Thus, the coverage depth decreases at the edge of the linear genome sequence when the reads are directly aligned to the sequence. Two experiments were performed to quantify the effect on growth rate estimation. First, growth rate estimates were computed and compared with the results obtained from a circularized sequence file. The circularized file was constructed by combining the head and tail portions in a FASTA file. Second, growth rate estimates were compared between Bowtie2 and vg, which can align a sequence on a graph, while also considering the circular structure of the genome. Both results showed significantly higher correlations; hence, we concluded that these procedures are

unnecessary to practically compute pPTR (Pearson r = 1.0, p-value < 0.001) (Supplementary Fig. 6).

8 *Distribution of the maximum continuous zero coverage size in the IBD dataset*

To estimate the maximum deletion size of the entire genome sequence in the human intestinal WGS reads relative to the reference sequence database, we quantified the size of the region in which the coverage depth was continuously 0. First, we aligned the WGS reads of the IBD dataset (Franzosa, 2018) to the complete genome sequence database using Bowtie2 and calculated the coverage depth with SAMtools. To distinguish between the coverage depths due to deletion and those due to an insufficient read amount, we focused only on the samples with average coverage depths greater than 10. Finally, we calculated the size of the maximum area in which the coverage depth was continuously 0.

9 *Approximation of zero coverage fraction with replication effects*

In previous studies, the zero coverage fraction has been modeled with the average coverage depth as a random variable, i.e., $\mathrm{E}(\widehat{f}) = \frac{1}{I}\sum_{i=1}^{I}\left(1-p_i\right)^{N_r}$, where $\widehat{f}$ is the rate of nucleotides with zero coverage depth, N_r is the number of reads, and other symbols follow those of our model (Roach, 1995). When the probability p_i is uniform, the function can be approximated as $\mathrm{E}(\widehat{f}) \approx \exp(-x)$, where x is the average coverage depth, in low average coverage and uniform probability (Lander & Waterman, 1988; Roach, 1995; Wendl & Waterston, 2002). We also investigated an approximation model to express the zero coverage fraction under the replication effect. Although it is not an analytical model, the theoretical zero-coverage fraction assuming the replication effect was approximated with the following equation with a minimum R^2 score of 0.98: $\mathrm{E}(\widehat{f}) \approx \exp(-b^{ax+1})$, where a and b are shape parameters of the function (Supplementary Fig. 11c). The theoretical score under the replication effects was calculated with the probability generated from the von Mises distribution. Then, we set 10,000 nt as sequence size I.

10 Simulation dataset to evaluate the performance of skewness detection

To evaluate the robustness of the extended model and asymmetricity to noise, we fitted the model to a simulated dataset and compared estimates of the model and *p*-values with the true parameter used to generate the data. For this purpose, a pseudo-coverage depth was constructed from InvMIAE von Mises and multinomial distributions. The length of the genome sequence was 1,000 nt, and depth was generated such that the total was 10,000. The skewness parameter was 0 for symmetric data and 0.5 for asymmetric data. Peak noise was generated at $-2/\pi$ with a length of 10 and depth of 0–45 coverage. For each peak noise, data were independently generated 10 times and fitting was performed. Although the estimate was slightly affected by noise due to the likelihood improvement, the asymmetry in the invMIAE model was properly inferred (Supplementary Figs. 16a and b). Next, robustness was examined in detail, where a dataset with artificial noise and a dataset with an artificial mutation in the 5,000 nt block size were used to verify the PTR robustness. Although the noise at the peak suppressed error with the optional procedure of excluding the top 1% depth (Supplementary Fig. 16c), it was sensitive to template sequence mutations (Supplementary Fig. 16d).

11 Determination of applicable threshold for asymmetric extended model

The robustness of the asymmetric extended model was determined based on the parameter q, which represents the probability of obtaining a non-zero in the zero-inflated negative binomial distribution. We quantified the probability in the previous dataset, as well as in the artificially mutated datasets. The parameter values estimated for both the *L. gasseri* and the *E. coli* datasets were greater than 0.99; however, it was estimated to be 0.896 in the E. *faecalis* dataset, and 0.952 and 0.859 for the *L. gasseri* datasets with 5% and 10% mutations, respectively (Supplementary Fig. 18a). Based on the evaluation of the robustness of the InvMIAE model (Supplementary Fig. 16d), it was decided that a q value greater than 0.95 is necessary to apply the asymmetric extended model.
